# Multiple Emission of Phosphonium Fluorophores Harnessed by the Pathways of Photoinduced Counterion Migration

**DOI:** 10.1002/anie.202115690

**Published:** 2022-02-24

**Authors:** Andrey Belyaev, Bo‐Kang Su, Yu‐Hsuan Cheng, Zong‐Ying Liu, Nasrulla Majid Khan, Antti J. Karttunen, Pi‐Tai Chou, Igor O. Koshevoy

**Affiliations:** ^1^ Department of Chemistry University of Eastern Finland Yliopistokatu 7 80101 Joensuu Finland; ^2^ Department of Chemistry National Taiwan University Taipei 106 Taiwan; ^3^ Department of Chemistry and Materials Science Aalto-University 00076 Aalto Finland

**Keywords:** Charge Transfer, Donor–Acceptor Systems, Dual Emission, Ion Pairs, Phosphonium

## Abstract

In the emerging field of intramolecular charge transfer induced counterion migration, we report the new insights into photophysical features of luminescent donor–acceptor phosphonium dyes (D−π−)_*n*_A^+^[X^−^] (π=−(C_6_H_4_)_*x*_−). The unique connectivity of the phosphorus atom affords multipolar molecules with a variable number of arms and the electronic properties of the acceptor group. In the ion‐paired form, the transition from dipolar to quadrupolar configuration enhances the low energy migration‐induced band by providing the additional pathways for anion motion. The multipolar architecture, adjustable lengths of the π‐spacers and the nature of counterions allow for efficient tuning of the emission and achieving nearly pure white light with quantum yields around 30 %. The methyl substituent at the phosphorus atom reduces the rate of ion migration and suppresses the red shifted bands, simultaneously improving total emission intensity. The results unveil the harnessing of the multiple emission of phosphonium fluorophores by anion migration via structure and branching of donor–acceptor arms.

## Graphical abstract

The photophysical properties of a series of multipolar donor–acceptor phosphonium fluorophores (D−π−)_*n*_A^+^[X^−^] have been investigated. These dyes exhibit steady‐state panchromatic luminescence attributed to the photoinduced counterion migration. The intra‐ion‐pair dynamics and the fluorescence response can be rationally tuned on the molecular level that might broaden the scope of functionalities and stimulate the development of new ionic systems.

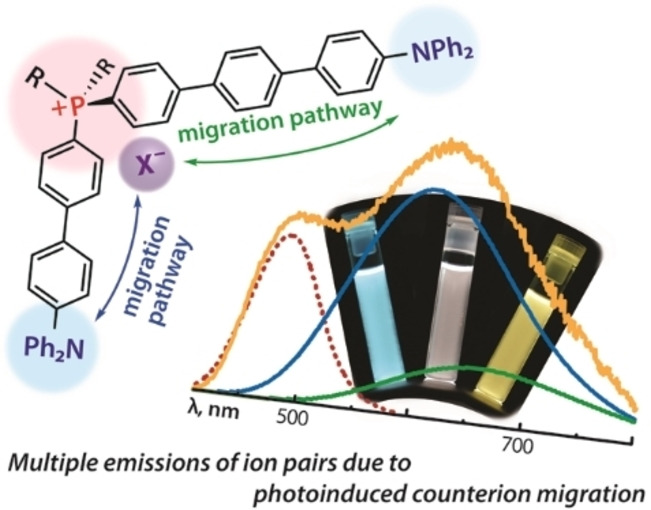

## Introduction

Light‐driven molecular motion is a fascinating property, which forms a basis for the development of unconventional materials,^[1]^ molecular switches, motors and machines.^[2]^ A major strategy to photo‐controlled movement of molecular components relies on the use of isomerizable organic units that ensures robustness of such photodynamic systems and simultaneously defines their functional behavior.^[3]^ Alternatively, the change of the electronic structure that occurs upon photoexcitation can perturb non‐covalent contacts between the constituents of supramolecular species and affect their mutual displacement.^[4]^ Thus, switching hydrogen‐bonding ability or electrostatic interactions by means of photoinduced electron transfer allowed one to realize fast (ca. 1–100 μs) translational motion in a rotaxane type of molecular shuttles.^[5]^ The employment of a photoacidic moiety capable of intermolecular proton transfer in a rotaxane structure allowed one to increase the rate of translocation on a distance of ca. 1 nm nearly by an order of magnitude.^[6]^ Even faster movement occurring on a picosecond time scale driven by excited‐state intramolecular charge transfer (ESICT) has been proposed for pyridinium donor–acceptor dyes embedded in the cucurbit[7]uril host.^[7]^ However, the relocations of the composite elements in these photo‐driven (pseudo)rotaxane shuttles typically generate very limited alterations of optical characteristics, which hardly can serve as convenient read‐out signals.

**Scheme 1:**
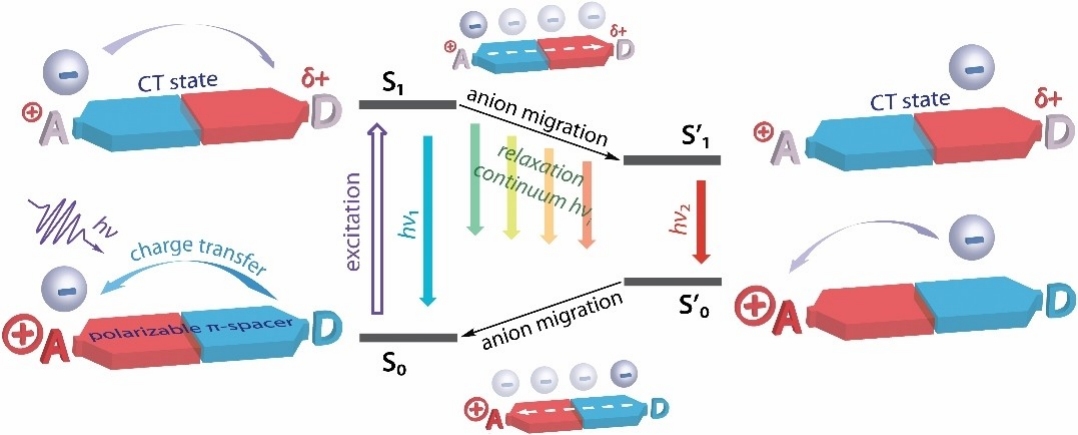
Schematic representation of ESICT‐induced anion migration in donor–acceptor ionic dyes in the absence of solvent relaxation (low polarity solvents).

The sensitivity of CT state of organic chromophores to the polarity of the local environment nevertheless potentially offers an attractive opportunity to design molecular systems, demonstrating motion‐produced optical function at high speed and in a reversible manner. For instance, it has been shown that photoexcitation of a donor–acceptor (D‐A) dye combined with a calixarene receptor leads to very fast translocation of a coordinated potassium ion within the host molecule, which is clearly reflected in time‐resolved absorption spectra.^[8]^ In the case of ionic D−π−A^+^ fluorophores, the CT‐modulated interactions within non‐dissociated ion pairs have been noticed to exhibit a more pronounced photophysical response.^[9]^ Recently, we reported structurally simple linear D−π−A^+^ fluorophores with phosphonium and pyridinium acceptor A^+^ units (D=−NR_2_ group, π=phenylene spacer),^[10]^ which reveal regular photoinduced CT and depletion of electron density from the donor motif. The redistribution of electron density and the formation of the −N^δ+^R_2_ site^[8]^ is stabilized in polar solvents by dipole–dipole interactions with a rapidly reorganized nearest shell of the medium. On the contrary, in solvents with low polarity, the photophysical behavior of these dyes is strongly influenced by the ion‐pairing effect. The latter is manifested by ESICT‐driven migration (or translocation) of a small counter anion from the cationic A^+^ area (ground state location) in the direction of the transient −N^δ+^R_2_ group (excited state target) that decreases the energy of the emissive state and leads to the evolution of a continuum of red‐shifted fluorescence bands (Scheme 1). The resulting dual‐band‐like steady‐state luminescence was shown to be dependent on the length of the π‐spacer, the size of migrating anion, and the solvent viscosity. Comparison of phosphonium^[10a]^ and pyridinium^[10b]^ congeners also indicate the important role of the cationic group and, consequently, polarization of the spacer in the process of light‐triggered anion migration and corresponding optical response.

In this emerging field, herein, we aim at exploring the factors affecting the ESICT‐induced ion motion and photophysical features of emissive ion‐paired D−π−A^+^ salts (π=−(C_6_H_4_)_*x*_−) via a new series of phosphonium dyes with various branching CT chromophores. The strategy lies in the unique connectivity and accessible quaternization of the phosphorus atom,^[11]^ which allow us to synthesize novel multipolar molecules (D−π−)_*n*_A^+^ with variable number of and length of linear donor–acceptor arms, ^+^PPh_4‐*n*_(−π−D)_*n*_ (*n*=1–4) and ^+^PPh_2_(−π−D)(−π′−D). To probe the cationic effect, we also chemically alter the acceptor group by introducing the methyl group along the aromatic substituents in ^+^PMePh_3‐*m*_(−π−D)_*m*_ (*m*=1, 3). The results elaborated below, for the first time, unveil the key role played by cation structure and donor–acceptor arms to the ESICT‐driven counterion migration and hence to the harnessing of the multiple emission.

## Results and Discussion

### Synthesis and Characterization

**Scheme 2:**
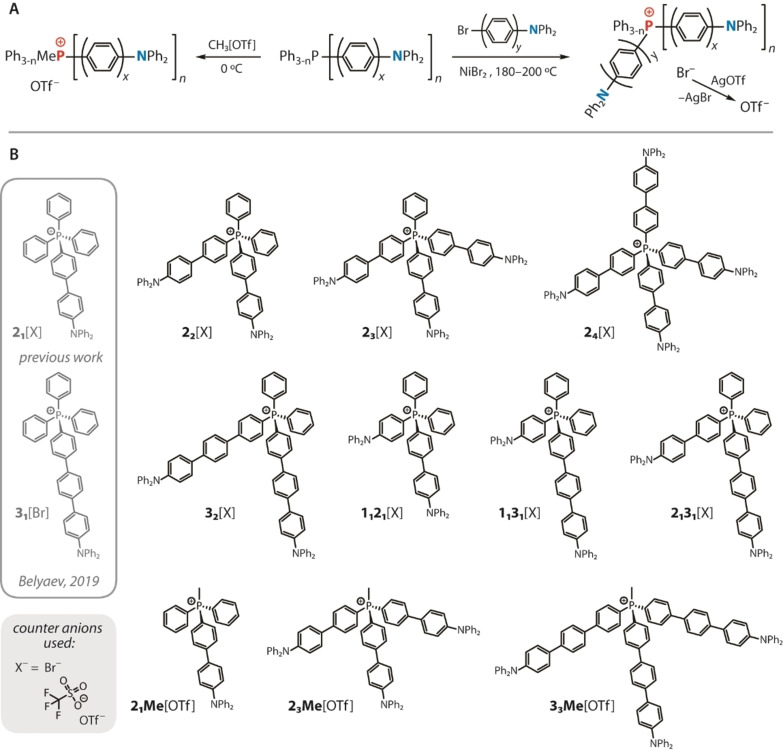
General synthetic routes (A) and structures of donor–acceptor phosphonium salts (B) studied in this work (named ***Y***_***n***_ ***Z***_***n*** **′**_[X], where ***Y*** and ***Z*** correspond to the length of phenylene linkers and the *n*, *n*′ subscripts indicate the number of the respective donor‐containing substituents).

The preparation of (D−π−)_*n*_A^+^[X^−^] phosphonium dyes is outlined in Scheme 2, where the experimental details are given in the Supporting Information. Following the general NiBr_2_‐catalyzed protocol,^[12]^ the reactions of appropriate donor‐functionalized phosphines PPh_3‐*n*_[−(C_6_H_4_)_*x*_−NPh_2_]_*n*_ (*x*=2, *n*=1 **L2_1_**,^[13]^ 3 **L2_3_**;^[14]^ *x*=3, *n*=1 **L3_1_**, 3 **L3_3_** ^[14]^) with the corresponding bromo‐*N,N*‐diphenyl‐aryl amines, Br−(C_6_H_4_)_*y*_−NPh_2_ (*y*=1–3),^[15]^ or bromobenzene (for **2_3_** [Br]) afford tetraaryl bromide salts, which were isolated as amorphous solids. These quaternized cationic species are designated as ***Y***_***n***_ ***Z***_***n*** **′**_[Br], where ***Y*** and ***Z*** correspond to the length of phenylene linkers and the *n*, *n*′ subscripts indicate the number of the respective donor‐containing substituents. Compounds **2_2_** [Br] and **2_3_** [Br] are obtained in good yields (77 and 80 %), and for the rest of the series the target compounds are generated with visibly smaller efficiency (34–52 %). The Pd‐catalyzed formation of phosphonium ions^[16]^ in this case is found to be less selective; for instance, the reactions of equimolar amounts of **L2_1_** and Br−(C_6_H_4_)_*y*_−NPh_2_ give mixtures of several P‐cationic products (Figure S1), which are difficult to separate. Whereas selectivity of the process can be significantly improved by lowering the temperature from 140 to ca. 115 °C, the overall conversion of the starting materials does not exceed 30 %. A phenol‐promoted metal‐free approach^[17]^ also delivers moderate to low yields (13 % for **2_2_** [Br] and 10 % for **2_3_** [Br]) because of limited solubility of the oligophenylene phosphine precursors **L2_1_** and **L2_3_**. Salts **2_1_** [X] and **3_1_** [Br] have been studied by us previously.^[10a]^ The metathesis of the counterion Br^−^→OTf^−^ was performed with silver(I) triflate in a nearly quantitative manner. Methylation of phosphines **L2_1_**, **L2_3_** and **L3_3_** was readily carried out with methyl triflate, giving salts **2_1_Me**[OTf], **2_3_Me**[OTf] and **3_3_Me**[OTf] in 83–93 % yield.

The composition of the products was confirmed by spectroscopic methods. The ESI^+^ mass spectra of all salts show one dominating signal that in each case corresponds to the molecular cation (Figure S2). The ^31^P, ^13^C and ^1^H NMR spectra (see the Supporting Information) are also compatible with the proposed structures (Scheme 2) and the presence of single species for each compound. Thus, the phosphorus resonances in CD_2_Cl_2_ appear in the ranges 22.3–23.4 ppm (aryl‐substituted ions) and 20.6–21.5 (methylated ions), which belong to a typical region for other acyclic phosphonium compounds.[^10a^, ^18^] The exchange of the anion shows virtually no effect on the chemical shift of the ^31^P signal in dichloromethane, indicating weak ion pair association in this solvent. In toluene‐d_8_, the NMR spectra are substantially more anion‐dependent, which can be seen in a non‐negligible difference in the δ(^31^P) for triflate and bromide salts (e.g. 21.6 and 22.4 ppm for **2_1_Me**[OTf] and **2_1_Me**[Br]; 21.1 and 20.7 ppm for **1_1_2_1_** [OTf] and **1_1_2_1_** [Br]). The corresponding proton spectra for the selected compounds (Supporting Information) also confirm a non‐innocent role of anion in ion pairing in low polarity medium, which has been demonstrated for the family **2_1_** [X].^[10a]^

### Photophysical Properties

To avoid complication, we segregate the dyes under study into three groups–*i*) compounds **2**_***n***_[X] where we probe the effect of branching the multipolar cations bearing identical substituents; *ii*) quadrupolar compounds containing two elongated π‐spacers **3_2_** [X], and two different spacers ***Y***_**1**_ ***Z***_**1**_[X] with competing migration pathways; *iii*) methylated species **2_1_Me**[OTf] and ***Y***_**3**_ **Me**[OTf] with sterically and electronically different quaternized phosphorus center.

### Polar Solvents

**Figure 1:**
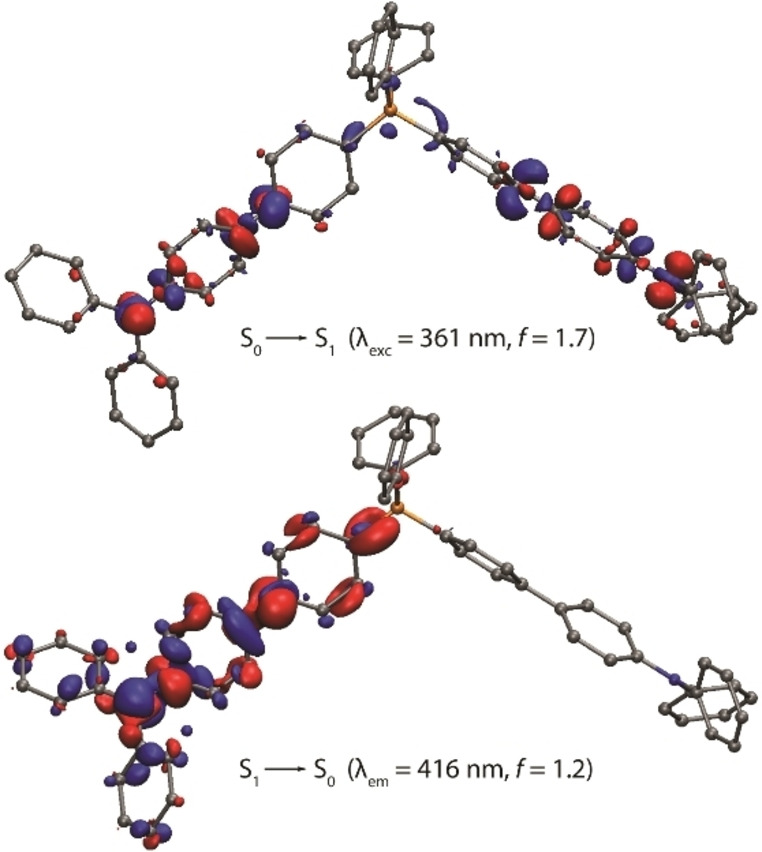
Lowest energy excitation S_0_→S_1_ and emission S_1_→S_0_ electron density difference plots for cation **2_2_** ^+^ (isovalue 0.002 a.u., DFT‐LRC‐ωPBEh method, optimized S_0_ and S_1_ geometries). During the electronic transition, the electron density increases in the blue areas and decreases in the red areas. Hydrogen atoms and counter anions are omitted for clarity.

In polar media such as CH_2_Cl_2_ and CH_3_CN one can neglect ion pairing effect due to efficient dissociation. In these solvents, optical properties of all titled salts are not exceptional and arise from intramolecular charge transfer (ICT) transitions expected from the D‐A architecture of these cations, being nearly independent on the counterion (Figures S3–S5, Table S1). The absorption profiles of **2**_***n***_[X] (X^−^=Br^−^, OTf^−^, Figure S3) are close to that of **2_1_** [X]^[10a]^ and display the lowest energy bands around 390–394 nm in CH_2_Cl_2_ (373–378 nm in CH_3_CN), where the molar attenuation coefficient additively grows with the increase of the number *n* of the −π−D arms. The structureless emission bands are almost identical for the entire series **2**_***n***_[X]. In addition, they all show positive solvatochromism, for which the peak wavelength shifts from 522–528 nm (Φ_em_=0.73–0.81) in CH_2_Cl_2_ to 547–551 nm (Φ_em_=0.25–0.39) in CH_3_CN with moderate to high emission quantum yields. Note that a small hypsochromic shift of the emission maxima for ca. 5 nm is identified within the series of **2**_***n***_^+^ (*n*=1→4) cations, which likely arises from a combination of slight decrease of electron‐accepting strength of the phosphonium unit and an additive effect of the donor‐containing substituents. This correlation is further supported by gradually decreasing Stokes shifts (from 6900 cm^−1^ for **2_1_** [Br] to 6288 cm^−1^ for **2_4_** [Br] in CH_2_Cl_2_, Table S1), and the chemical shifts in the ^31^P NMR spectra; the latter indicate slight shielding of the phosphorus nuclei from *n*=1 to *n*=4 (23.5 ppm for **2_1_**, 23.2 ppm for **2_2_**, 22.9 ppm for **2_3_**, 22.6 ppm for **2_4_**). Very close fluorescence spectral properties of **2**_***n***_[X] in dichloromethane and acetonitrile suggest that the corresponding electronic transitions mostly occur within one {^+^P−(C_6_H_4_)_2_−NPh_2_} push‐pull arm, i.e. there is no appreciable electronic coupling between A^+^−D subchromophores in A^+^(−D)_*n*_ (*n*=2–4) multipolar ions, which can be approximated in polar solvents as chromophores with dipolar symmetry‐broken excited state.^[19]^ Optimization of the S_0_ geometry for the cation **2_2_** ^+^ with density functional theory (DFT) leads in a geometry where both biphenylene spacers are somewhat twisted with estimated torsion angle of about 30°. The lowest energy absorption S_0_(optimized)→S_1_ in **2_2_** ^+^ involves two equivalent chromophore arms and results in substantial charge transfer from the donor −NPh_2_ motifs as well as changing polarization of the π‐spacer (Figure 1). At the optimized S_1_ geometry, one −(C_6_H_4_)_2_−NPh_2_ fragment becomes flattened and is responsible for the emission that makes **2_2_** ^+^ behaving in a dipolar manner.

In CH_2_Cl_2_ and CH_3_CN, the behavior of salts **3_2_** [X], **1_1_3_1_** [X] and **2_1_3_1_** [X] (X^−^=Br^−^, OTf^−^) is dominated by the −π−D fragment with terphenylene spacer (Figure S4 and Table S1). The non‐symmetric cations **1_1_3_1_** ^+^ and **2_1_3_1_** ^+^ reveal minor hypsochromic shifts of yellow‐orange emission, and substantially higher quantum yields (Φ_em_=0.77/0.66 in CH_2_Cl_2_, 0.32/0.15 in CH_3_CN) than **3_2_** ^+^ congener (Φ_em_=0.30 in CH_2_Cl_2_, 0.06 in CH_3_CN). The lack of dual fluorescence for **1_1_3_1_** ^+^ and **2_1_3_1_** ^+^ in polar media is in line with Kasha's rule, i.e. the emission is associated with the dipolar lowest excited state, which corresponds to the ICT occurring on the arm with the longest terphenylene spacer. The DFT‐calculated electronic transitions S_0_→S_1_/S_0_←S_1_ in cation **2_1_3_1_** ^+^ obtained for symmetry unconstrained geometries (*C*_1_) primarily involve the shorter chromophore component, which becomes flattened during the geometry optimization of the S_1_ state (Figure S5). However, considering an average conformation (*C_s_*) with flattened phenylene spacers on both chromophores shifts the localization of electronic transitions related to the excitation/emission mainly to the terphenylene fragment. The *C_s_* structure is energetically higher than the *C*_1_ local minimum by only 10 kJ mol^−1^ per phenylene group, making the *C_s_* structure an energetically feasible idealized description of the molecular conformation in solution.

The absorption and emission signals of the methylated dyes **2_1_Me**[OTf], **2_3_Me**[OTf] and **3_3_Me**[OTf] in CH_2_Cl_2_ and CH_3_CN demonstrate modest hypsochromic shifts with respect to their tetraarylated relatives (Figure S6 and Table S1). Apparently, this energy increase, which is more pronounced in more polar acetonitrile, is caused by non‐electron accepting methyl substituent and therefore less polarizing effect of the phosphonium group.

### Non‐polar Solvents

**Figure 2:**
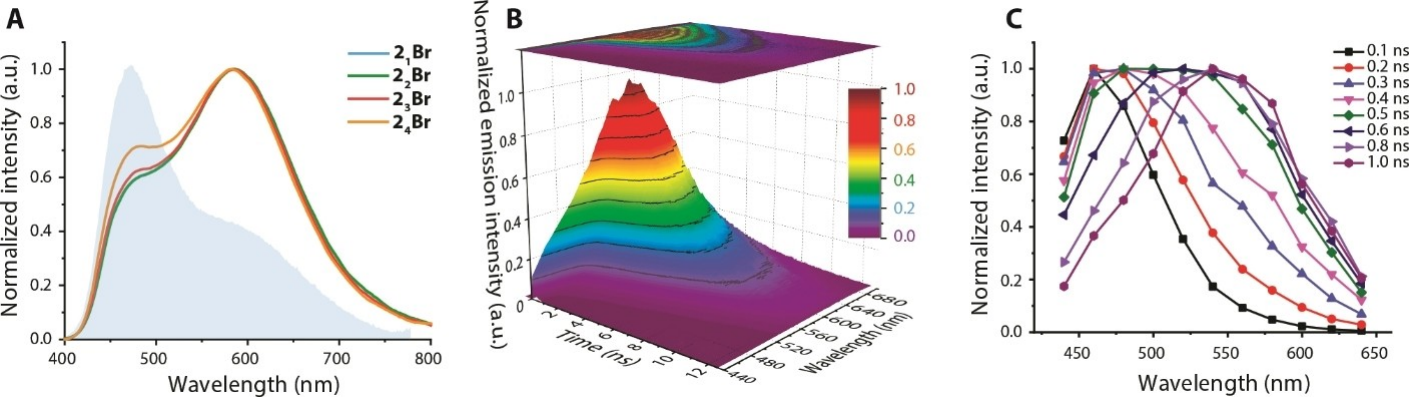
A) Normalized emission spectra of **2**_***n***_[Br] (filled graph corresponds to **2_1_** [Br]^[10a]^). B), C) Time‐resolved emission spectra of **2_2_** [OTf] in toluene at 298 K.

*i*) *Multipolar dyes **2***_***n***_ *[X] (n*=*2–4; X* ^*−*^=*Br^−^, OTf^−^)* In the lower polarity solvent such as toluene, where ion pairing is anticipated, branching the molecular structure of the phosphonium cations has a distinct influence on the photoluminescence characteristics. In accordance with earlier studies,^[10a]^ new fluorophores **2**_***n***_[X] demonstrate high‐energy (HiE, F_1_) and low‐energy (LoE, F_2_) emission bands of variable ratio in their steady‐state spectra (Table 1, Figures 2 and S7). The excitation spectra recorded for F_1_ and F_2_ emission bands for all **2**_***n***_[X] (Figure S8) are identical with the absorption profiles, confirming that they both share common ground‐state species. By analogy with **2_1_** ^+^, the F_1_ band is assigned to an anion‐destabilized ICT state S_1_, whereas the red‐shifted F_2_ band emerges upon photoinduced anion migration and consequent stabilization of the initial charge separated state (S_1_′, Scheme 1). The emission spectra of **2_2_** [OTf] in frozen toluene and dichloromethane solutions at 77 K display only the blue shifted HiE signals (Figure S9). The results imply completely suppressed anion migration in frozen ion pair, which is electrostatically stabilized in the ground state but destabilized in the CT excited state.

**Table 1:** Photophysical data of the studied phosphonium salts in toluene at 298 K.

Cation	Anion A^−^	*λ*_abs_ [nm]	*λ*_em_ [nm]	*F*_1_/*F*_2_^[a]^	Φ_em_	*τ*_F1_ [ps] (prefactor)^[b]^	*τ*_F2_ [ps] (prefactor)^[b]^	CIE
**2_1_** ^[10a]^	Br^−^	379	468, 592sh	1/0.41	0.17	67 (0.63), 450 (0.37)	93 (−0.49), 792 (0.36), 2439 (0.15)	0.29, 0.34
	OTf^−^	383	468, 589	0.39/1	0.47	150 (0.73), 660 (0.27)	298 (−0.45), 3807 (0.55)	0.42, 0.43
**2_2_**	Br^−^	380	468, 586	0.57/1	0.15	70 (0.95), 350 (0.05)	95 (−0.51), 648 (0.36), 2058 (0.13)	0.39, 0.41
	OTf^−^	385	468, 586	0.29/1	0.38	111 (0.99), 2741 (0.01)	185 (−0.51), 3306 (0.49)	0.42, 0.45
**2_3_**	Br^−^	382	468, 584	0.60/1	0.15	71 (0.99), 605 (0.01)	97 (−0.48), 610 (0.33), 1983 (0.19)	0.39, 0.41
	OTf^−^	386	468, 584	0.30/1	0.34	90 (0.99), 2362 (0.01)	163 (−0.51), 2904 (0.49)	0.43, 0.45
**2_4_**	Br^−^	383	468, 582	0.69/1	0.10	65 (0.98), 1037 (0.02)	112 (−0.50), 550 (0.31), 1669 (0.19)	0.38, 0.40
	OTf^−^	386	468, 580	0.35/1	0.30	79 (0.99), 2285 (0.01)	171 (−0.49), 2627 (0.51)	0.42, 0.44
**3_1_**	Br^−[10a]^	373	480, 660	1/0.18	0.17	120 (0.39), 780 (0.61)	120 (−0.39), 2540 (0.61)	0.21, 0.29
	OTf^−^	372	482, 661	1/0.64	0.42	80 (0.52), 782 (0.48)	405 (−0.38), 3600 (0.62)	0.31, 0.34
**3_2_**	Br^−^	375	480, 665	1/0.29	0.19	118 (0.36), 470 (0.64)	118 (−0.46), 1971 (0.64)	0.24, 0.30
	OTf^−^	375	481, 664	1/0.84	0.29	93 (0.37), 466 (0.63)	290 (−0.46), 3433 (0.54)	0.34, 0.34
**1_1_2_1_**	Br^−^	370	470, 570	1/0.80	0.17	86 (0.74), 500 (0.25), 2798 (0.01)	105(−0.51), 938 (0.27), 3385(0.22)	0.32, 0.35
	OTf^−^	371	470, 576	0.60/1	0.53	93 (0.55), 637 (0.43), 3381 (0.02)	424 (−0.47), 4043 (0.53)	0.38, 0.41
**1_1_3_1_**	Br^−^	361	473, 645	1/0.30	0.21	115 (0.73), 761 (0.27)	90 (−0.50), 1520 (0.27), 2694 (0.23)	0.25, 0.28
	OTf^−^	361	471, 642	1/0.55	0.49	85 (0.53), 1012 (0.47)	520 (−0.44), 3908(0.56)	0.30, 0.31
**2_1_3_1_**	Br^−^	377	477, 615	1/0.48	0.09	65 (0.55), 237 (0.44), 1536 (0.01)	121 (−0.49), 769 (0.27), 1806 (0.24)	0.29, 0.32
	OTf^−^	378	480, 613	0.66/1	0.23	65 (0.45), 248 (0.54), 2707 (0.01)	197 (−0.50), 3262 (0.50)	0.40, 0.40
**2_1_Me**	OTf^−^	378	465, 583	1/052	0.53	604 (0.87), 3477 (0.13)	657 (−0.49), 3602 (0.51)	0.29, 0.31
**2_3_Me**	OTf^−^	380	461, 573	1/0.55	0.62	439 (0.89), 2700 (0.11)	643 (−0.49), 3225 (0.51)	0.29, 0.31
**3_3_Me**	OTf^−^	371	473, 650	1/0.12	0.79	1088 (0.95), 2350 (0.05)	1496 (−0.44), 3264 (0.56)	0.20, 0.27

[a] The F_1_/F_2_ ratio was calculated using the intensities of the emission spectra at the maxima of the F_1_ and F_2_ bands. [b] Measured at the 440 (*τ*_F1_) and 640/660 nm (*τ*_F2_) for **2**/**3** containing cations.

Due to higher stability, triflate salts were used for most of time‐resolved studies. Transient emission spectra monitored in the 0.1–1.0 ns time range for **2**_***n***_[OTf] (*n*=2–4; Figures 2 and S7) reveal that the initial HiE F_1_ band, recorded at 0.1 ns delay after the excitation pulse, undergoes continuous transformation into the LoE F_2_ fluorescence, which is completed within 0.8–1.0 ns. The spectral temporal evolution of red‐shifted emission correlates well with the kinetics of the F_2_ band, for which a rise component of ca. 170 ps can be resolved by time‐correlated single photon counting (TCSPC) measurements, followed by the relaxation of the excited state with *τ*_obs_=2.6–3.3 ns (Table 1, Figure S9).

**Figure 3:**
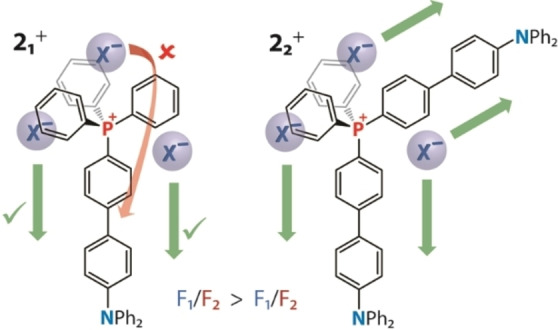
Schematic representation of the effect of dipolar and quadrupolar architectures on the possibility of anion migration.

Triflate salts demonstrate systematically higher quantum yields (Φ_em_=0.47–0.30) than the bromide congeners (Φ_em_=0.17–0.10), while the multipolar dyes show somewhat lower intensity than dipolar compounds. The transition from dipolar (D−A^+^) **2_1_** ^+^ to quadrupolar (D−A^+^−D) **2_2_** ^+^ architecture in the presence of Br^−^ anion dramatically increases the intensity of F_2_ band (*λ*=586 nm) relative to F_1_ band (*λ*=468 nm) that changes the F_1_/F_2_ ratio from 1/0.41 to 0.57/1 (Figure 2 and Table 1) but retains both the emission wavelengths and the total quantum yields (Φ_em_=0.17 and 0.15 for **2_1_** [Br] and **2_2_** [Br]). Qualitatively, the same trend is observed for OTf^−^ salts, for which the LoE signal dominates and grows for **2_2_** ^+^ vs **2_1_** ^+^ cation (Figure S7). Schematically, it can be illustrated by the symmetrically branched molecular structure of **2_2_** ^+^ that increases the probability of photoinduced anion translocation by providing two accessible migration pathways and minimizing steric restrictions, i.e. eliminating migration‐unfavorable locations for relatively small counterions around the cationic motif (Figure 3). An additional enhancement of migration can be offered by the quadrupolar excited state, which might have an appreciable contribution in non‐polar solvents.^[19d]^

Further addition of the subchromophores to the phosphorus atom in **2_3_** ^+^ and **2_4_** ^+^ slightly raises both the emission energy of the F_2_ band, and the intensity of the F_1_ band (Figures 2A and S7A, Table 1). The subsequent increase of the number of equivalent −π−D arms in *C*_3_ and *T* symmetric **2_3_** ^+^ and **2_4_** ^+^ is not expected to improve significantly steric conditions for anion migration compared to those in **2_2_** ^+^. This supposition correlates with close rise times for the F_2_ band among multipolar **2**_***n***_[OTf] species (*n*=2–4, with a rise component of τ_F2_ of 163–185 ps). Possible delocalization of the CT excited state over three (**2_3_** ^+^) and four (**2_4_** ^+^) sites might somewhat decrease the depletion of electron density from the NPh_2_ groups with respect to dipolar compounds. This should disfavor the Coulomb attraction X^−^⋅⋅⋅ ^δ+^NPh_2_ in the charge‐separated states (Scheme 1 and Figure 3), and therefore slightly diminish the efficiency of anion migration and the intensity of F_2_ band.

**Figure 4:**
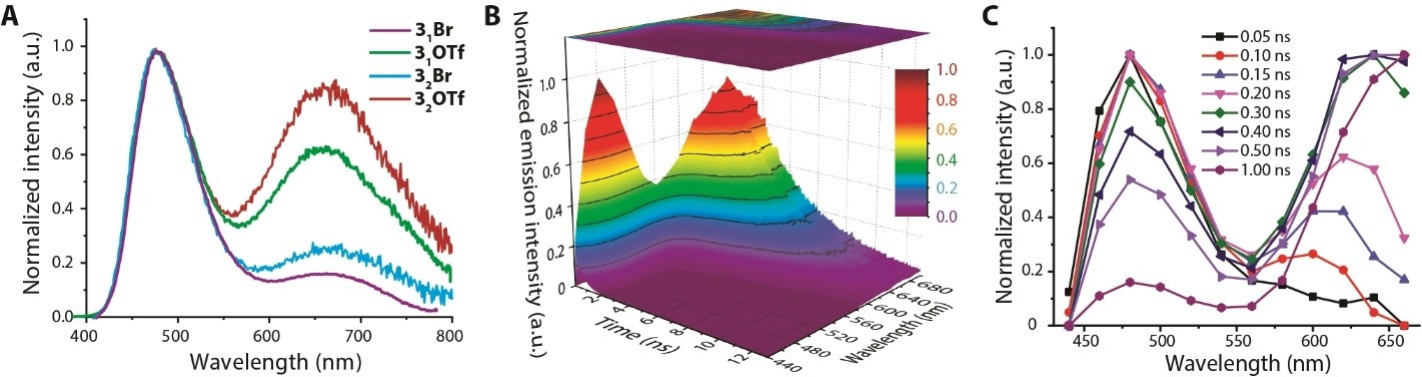
A) Normalized emission spectra of **3**_***n***_[X] (*n*=1, 2; A^−^=Br^−^, OTf^−^). B), C) Time‐resolved emission spectra of **3_2_** [OTf] in toluene at 298 K.

*ii*) *Quadrupolar dyes **3***_***2***_ *[A], **1**_1_ **2***_***1***_ *[A], **1***_***1***_ ***3***_***1***_ *[A], **2***_***1***_ ***3***_***1***_ *[A] (A* ^*−*^=*Br^−^, OTf^−^)* Taking into account that major alterations of luminescence for **2**_***n***_^+^ series are observed between the *a*) dipolar and quadrupolar ions, and *b*) different counterions, in the next stage we explore the tunability of the emission among the quadrupolar D−A^+^−D and D−A^+^−D′ type of dyes. As we have shown for salts **2_1_** [Br] and **3_1_** [Br],^[10a]^ elongation of the π‐spacer from biphenylene to terphenylene insignificantly red shifts the F_1_ band (from 468 to 480 nm), but has a more drastic effect on the F_2_ signal, increasing its wavelength from 592 to 660 nm that is accompanied by a drop of its intensity to produce sky‐blue fluorescence. Quadrupolar configuration of **3_2_** [Br] enhances the F_2_ band (the F_1_/F_2_ ratio varies from 1/0.18 for **3_1_** [Br] to 1/0.29, Figure 4). In line with the behavior of **2**_***n***_[X], the intensity of the LE band for **3_2_** ^+^ demonstrates further growth upon exchange of the Br^−^ for the OTf^−^ counterion (F_1_/F_2_=1/0.84 for **3_2_** [OTf]). This results in nearly pure white light emission demonstrated by **3_2_** [OTf], Commission Internationale de l'Eclairage (CIE) coordinates (0.34, 0.34), with good quantum yield of 0.29 (Table 1).

The hypothesis of photoinduced anion migration that is responsible for temporal growth of the LoE band, initially elaborated for **2**_***n***_^+^ series, is applicable to the dyes with longer terphenylene π‐spacers. Similar to **2**_***n***_^+^, for the **3**_***n***_^+^ series, the identity of the excitation spectra monitored in the range of the emission from 450 to 700 nm for salts containing −(C_6_H_4_)_3_−NPh_2_ fragment (Figure S10) points to the common origin of the F_1_ and F_2_ fluorescence bands. Time‐resolved data (Figures 4 and S11) also fit well the proposed concept and confirm the rise of red shifted F_2_ signal that follows the initially developed HiE band.

**Figure 5:**
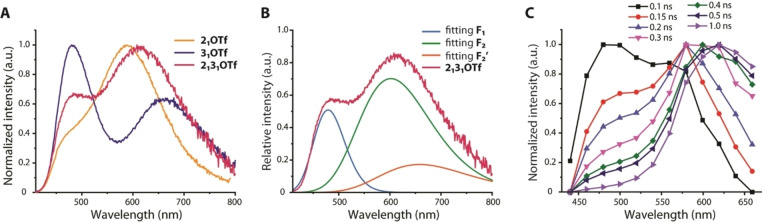
A) Comparison of normalized emission spectra of **2_1_** [OTf], **3_1_** [OTf] and **2_1_3_1_** [OTf]. B) Simulated deconvolution of the emission spectrum of **2_1_3_1_** [OTf] into three components (one HiE F_1_ and two migration‐induced F_2_ and F_2_′ bands). C) Time‐resolved emission spectra of **2_1_3_1_** [OTf]; toluene, 298 K.

Combination of two different chromophores in cations **1**_1_ **2_1_** ^+^, **1_1_3_1_** ^+^ and **2_1_3_1_** ^+^ has little influence on their emission in polar solvents compared to that of individual species **2_1_** ^+^ and **3_1_** ^+^ (see above, Figures S3 and S4), which is dominated by the longest −π−D fragment. Contrarily, in toluene these asymmetric dyes exhibit the contribution of both components into the total steady state luminescence (Figures S12–S14). For detailed analyses we have chosen salt **2_1_3_1_** [OTf], the emission spectrum of which is shown in Figure 5. Its spectral profile suggests the presence of two active migration pathways along two different −π−D arms and may be seen as a superposition of those of dipolar dyes **2_1_** [OTf] and **3_1_** [OTf]. Due to similarity of HiE bands of the latter compounds (468 and 482 nm), the experimental spectrum of **2_1_3_1_** [OTf] can be fitted with three components, which correspond to the average F_1_ and two migration‐induced LoE bands (F_2_ and F_2_′) assigned to the −biphenylene−NPh_2_ (**2**) and −terphenylene−NPh_2_ (**3**) motifs (Figure 5B). Time‐dependent emission spectra of **2_1_3_1_** [OTf] (Figure 5C) also support this process of competing migration, and clearly display the appearance of the intermediate F_2_ signal around 575 nm, which occurs in a faster time scale than F_2_′and is absent for **3**_***n***_[OTf] congeners (Figure 4).

The possibility of such anion motion in two non‐equivalent directions in the ion pair of **2_1_3_1_** [OTf] is tentatively ascribed to the excitation of both constituting chromophores having close energies (Figure S5). Possible delocalization of the S_1_ state over the entire cation or the contribution of two dipolar excited configurations (i.e. 2_1_*3_1_ ^+^ and 2_1_3_1_* ^+^) should provide two opportunities for anion migration and therefore lead to the evolution of two red shifted bands, F_2_ and F_2_′. Two other non‐symmetrical salts, **1**_1_ **2_1_** [OTf] and **1_1_3_1_** [OTf], generally operate pursuant to **2_1_3_1_** [OTf]. Their LoE bands are slightly blue shifted relative to **2_1_** [OTf] and **3_1_** [OTf], respectively (Figure S12). The short −phenylene−NPh_2_ (**1**) group, the emission of which is poorly responsive to anion migration,^[10a]^ evidently causes the growth of the F_1_ signal in **1**_1_ **2_1_** [OTf] and **1_1_3_1_** [OTf]. On the other hand, in contrast to **2_2_** [OTf] (Φ_em_=0.38), **2_1_3_1_** [OTf] (Φ_em_=0.23) and **3_2_** [OTf] (Φ_em_=0.29), branching the dipolar phosphonium ions with chromophore **1** does not have a detrimental effect on the quantum yields, which in the case of **1**_1_ **2_1_** [OTf] (Φ_em_=0.53) and **1_1_3_1_** [OTf] (Φ_em_=0.49) slightly exceed those of **2_1_** [OTf] (Φ_em_=0.47) and **3_1_** [OTf] (Φ_em_=0.42, Table 1).

**Figure 6:**
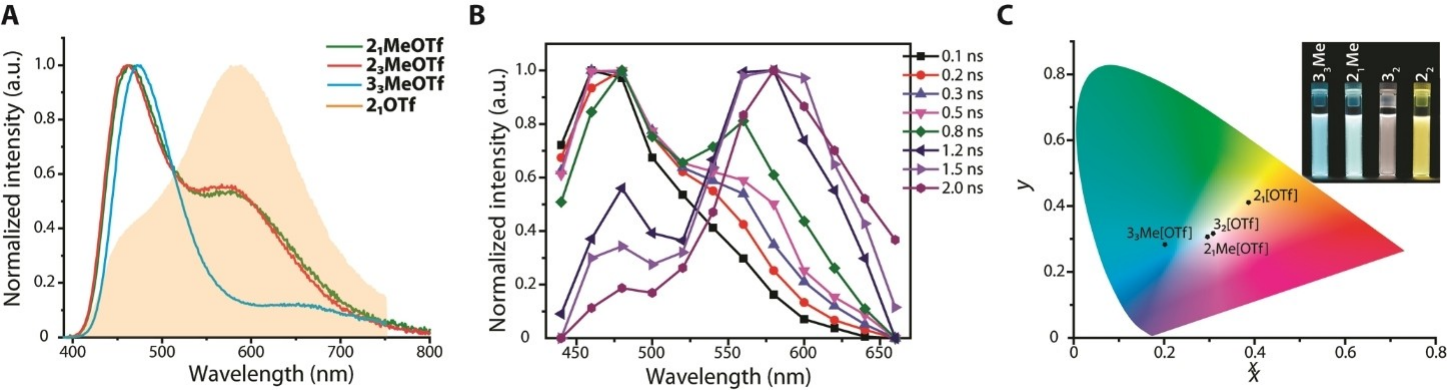
A) Normalized emission spectra of **2_1_Me**[OTf], **2_3_Me**[OTf] and **3_3_Me**[OTf] (filled graph corresponds to **2_1_** [OTf]^[10a]^). B) Time‐resolved emission spectra of **2_1_Me**[OTf]. C) CIE 1931 coordinates for **2_1_** [OTf], **2_1_Me**[OTf], **3_2_** [OTf] and **3_3_Me**[OTf]; toluene, 298 K.

*iii*) *Methylated dyes **2***_***1***_ ***Me**[OTf], **2***_***3***_ ***Me**[OTf] and **3***_***3***_ ***Me**[OTf]*. Tuning the electron deficient nature of the phosphonium group and its polarizing ability toward the −π−D motifs envisages the variation of the environment around the phosphorus atom. In this approach, the methylated derivatives **2_1_Me**[OTf], **2_3_Me**[OTf] and **3_3_Me**[OTf] were selected to probe the effect of electronic and steric features of the −^+^PR_3_ moiety on their optical properties. Accordingly, the electron deficiency of the phosphonium group can be directly identified by the chemical shift from the ^31^P NMR spectrum (see above). The replacement of the phenyl in cations **2_1_** ^+^ and **2_3_** ^+^ for the electron‐donating methyl substituent leads to upfield shift of the phosphorus resonances from 22.3–23.4 ppm (tetraaryl‐substituted ions) to 20.6–21.5 ppm (methylated ions). Surprisingly, this simple modification of the phosphonium group transforms yellowish emission of the tetraaryl triflates **2_1_** [OTf] and **2_3_** [OTf] with dominating LoE band (F_1_/F_2_=0.39/1 and 0.30/1, CIE 0.42,0.43 and 0.43,0.45) into cold white‐blueish luminescence of the methylated congeners **2_1_Me**[OTf] and **2_3_Me**[OTf] with predominant HiE band (F_1_/F_2_=1/0.52 and 1/0.55, CIE 0.29,0.31) (Figure 6). Further, the largest quantum yield (Φ_em_=0.62) is achieved for multipolar configuration of **2_3_Me**[OTf], which is opposite to arylated cations exhibiting the highest intensities among the dipolar compounds.

The time‐resolved spectra (Figures 6B and S15) unequivocally indicate considerably slower evolution of F_2_ band for **2_1_Me**[OTf] and **2_3_Me**[OTf] that correlates with much longer rise times of this LoE signal (657 and 643 ps, Figure S16 and Table 1) in comparison with those of **2_1_** [OTf] and **2_3_** [OTf] (298 and 163 ps, Table 1). Within the framework of our hypothesis, such spectroscopic features propound less efficient anion migration for methyl‐containing cations. Tentatively, we attribute the phenomenon to the combination of steric and polarization effects induced by the methyl group. In the S_0_ state (no solvation model applied), the dipole moment of **2_1_Me** ^+^ (13.8 D) is visibly larger than that of **2_1_** ^+^ (11.9 D). This is in line with ca. 30 kJ mol^−1^ higher ion pairing energy predicted for the optimized structures of **2_1_Me**[OTf] and **2_2_** [OTf]. In the excited S_1_ state, the dipole moment of **2_1_** ^+^ and **2_1_Me** ^+^ decreases to 1.7 D and 4.3 D, respectively. This implies a somewhat smaller charge redistribution for **2_1_Me** ^+^ compared to that for **2_1_** ^+^ in the S_1_ state and could result in a less favourable anion migration along the **2_1_Me** ^+^ cation. Consequently, the system undergoes radiative relaxation primarily from charge destabilized S_1_ state (Scheme 1) producing predominant HiE fluorescence. The smaller bromide anion in **2_1_Me**[Br] reveals further decrease in the intensity of the F_2_ band (Figure S19), which generally supports the hypothesis. We have optimized the S_0_ geometry for salts **2_1_Me**[Br] and **2_1_** [Br], see Supporting Information. In both cases the bromide anion shows attractive interactions with the H atoms of the phenyl or methyl fragments, which seem to be stronger for H(Me)⋅⋅⋅Br contact thanks to higher polarization of the Me. These secondary interactions remain in the CT state and further hinder the movement of the anion for methylated cations. The terphenylene‐based compound **3_3_Me**[OTf] adheres the same tendency, manifested by a substantially decreased intensity of the F_2_ band (F_1_/F_2_=1/0.12, CIE 0.20, 0.27) and additional growth of its rise time as long as 1496 ps.

## Conclusion

In summary, via utilizing the unique organophosphonium motif, which chemically combines versatile connectivity of the quaternized phosphorus atom with pronounced electron‐deficient properties, we have prepared a series of multipolar donor–acceptor ionic dyes (D−π−)_*n*_A^+^[X^−^] (D=NPh_2_, A^+^=^+^PR_4−*n*_ derivative) to investigate the effect of molecular structure on the photophysical behavior in relation to the counterion migration proposed for non‐dissociated ion pairs in solvents of low polarity. For this purpose, we varied the amount of the constituting chromophores around the P center ^+^PPh_4−*n*_(−π−D)_*n*_ (*n*=1–4, π=−(C_6_H_4_)_*x*_−), combined two different donor–acceptor arms ^+^PPh_2_(−π−D)(−π′−D) in the same cation, and changed the steric and electronic features of the phosphonium fragment in methylated species ^+^PMePh_3−*m*_(−π−D)_*m*_ (*m*=1, 3). In polar solvents (dichloromethane, acetonitrile), the new compounds reveal regular fluorescence with positive solvatochromism, virtually independent on the branching of the dye cation and assigned to dipolar‐like intramolecular charge transfer (ICT) excited state, which originates primarily from the −π−D component with the largest π‐spacer. In lower polarity medium (toluene), all salts are expected to exist as ion pairs and exhibit spectral temporal evolution of multiple emissions shifting from blue to red region. The resulting steady‐state panchromatic luminescence is associated with continuous electrostatic stabilization of the excited state upon anion migration from the ground‐state location in the direction opposite to that of ICT. In the ion paired form, the transition from dipolar to quadrupolar molecular structure substantially enhances the low energy band due to stereochemical reasons by providing the additional migration pathways. The quadrupolar architecture together with the adjustable lengths of the π‐spacers, and the nature of counterions allow for efficient tuning of the emission and achieving nearly pure white light with appreciable quantum yields. On the other hand, introducing the methyl substituent to the phosphorus atom dramatically reduces the rate of ion migration and suppresses the red shifted bands simultaneously improving total emission intensity. The presented results thus point to a quite general character of fast photoinduced anion motion that occurs in ion‐paired dyes and is accompanied by the optical response. This multiple intra‐ion‐pair dynamics and the corresponding fluorescence response can be rationally fine‐tuned on the molecular level that might considerably broaden the scope of functionalities and stimulate the development of new ionic systems for molecular machinery.

## Experimental Section

Synthetic, other experimental and computational details (PDF) are given in the Supporting Information.

## Conflict of interest

The authors declare no conflict of interest.

1

## Supporting information

As a service to our authors and readers, this journal provides supporting information supplied by the authors. Such materials are peer reviewed and may be re‐organized for online delivery, but are not copy‐edited or typeset. Technical support issues arising from supporting information (other than missing files) should be addressed to the authors.

Supporting Information

Supporting Information

## Data Availability

The data that support the findings of this study are available in the Supporting Information of this article.
