# Supplementary material for: Multiple Emission of Phosphonium Fluorophores Harnessed by the Pathways of Photoinduced Counterion Migration
Source: Angew Chem Int Ed Engl. 2022 Feb 24;61(19):e202115690. doi: 10.1002/anie.202115690 (PMC9306779; doi:10.1002/anie.202115690)
Supplement: Supplementary file 1 — Supporting Information [file ANIE-61-0-s001.pdf]

Angewandte Chemie
Eine Zeitschrift der Gesellschaft Deutscher Chemiker

Supporting Information

Multiple Emission of Phosphonium Fluorophores Harnessed by the Pathways of Photoinduced Counterion Migration

A. Belyaev, B.-K. Su, Y.-H. Cheng, Z.-Y. Liu, N. M. Khan, A. J. Karttunen*, P.-T. Chou*, I. O. Koshevoy**

Table of Contents

Experimental procedures	3
Figure S1. ^{31}P NMR spectra of Pd-catalyzed reactions of **L2_1**.	8
Figure S2. ESI$^+$ MS of salts ***Y_nZ_n***[X] and ***Y_n*Me**[X].	9
Table S1. Photophysical data of ***Y_nZ_n***[X] and ***Y_n*Me**[X] salts in DCM and MeCN.	11
Figure S3-S4. Absorption and emission spectra of salts **2_n**[X], **1_12_1**[OTf], **1_13_1**[OTf], **2_13_1**[OTf] and **3_2**[OTf] in DCM and MeCN.	12
Figure S5. Lowest energy excitation $S_0 \rightarrow S_1$ and emission $S_1 \rightarrow S_0$ electron density difference plots for cation **$2_13_1^+$**.	13
Figure S6. Absorption and emission spectra of salts **2_1Me**[OTf], **2_3Me**[OTf] and **3_3Me**[OTf] in DCM and MeCN.	13
Figure S7. Normalized emission spectra of **2_n**[OTf], time-resolved emission spectra of **2_3**[OTf] and **2_4**[OTf] in toluene.	14
Figure S8. Absorption and excitation spectra of **2_n**[OTf] in toluene at 298 K.	14
Figure S9. Emission spectra of 2_2[OTf] in frozen glass of toluene and DCM, and in solid state.	14
Figure S10. Emission decay profiles of 2_n[OTf] monitored at 440 nm and 640 nm in toluene at 298 K.	15
Figure S11. Normalized absorption and excitation spectra of **1_12_1**[OTf], **1_13_1**[OTf], **2_13_1**[OTf] and **3_2**[OTf] in toluene.	15
Figure S12. Emission decay profiles of **3_1**[OTf], **3_2**[Br] and **3_2**[OTf] in toluene.	16
Figure S13. Emission spectra of **1_12_1**[OTf], **1_13_1**[OTf] and **2_13_1**[OTf] in toluene.	16
Figure S14. Time-resolved emission spectra of **1_12_1**[OTf] and **1_13_1**[OTf] in toluene.	17
Figure S15. Emission decay profiles of **1_12_1**[OTf], **1_13_1**[OTf], **2_13_1**[OTf] in toluene.	17
Figure S16. Time-resolved emission spectra of **2_3Me**[OTf] and **3_3Me**[OTf] in toluene.	18
Figure S17. Emission decay profiles of **2_1Me**[OTf], **2_3Me**[OTf] and **3_3Me**[OTf] in toluene.	18
Figure S18. Absorption and excitation spectra of **2_1Me**[OTf], **2_3Me**[OTf] and **3_3Me**[OTf] in toluene.	19
Figure S19. Excitation and emission spectra of 2_1Me[Br] in toluene, and emission spectra of 2_1Me[Br] and 2_1Me[OTf] in toluene.	19
NMR spectra	20
References	28

Experimental Procedures

General comments

4-bromo-(*N,N*-diphenyl)-aniline, 4-(*N,N*-diphenylamino)-benzeneboronic acid,[1] 4-bromo-4´-(*N,N*-diphenylamino)-biphenyl, 4-bromo-4´´-(*N,N*-diphenylamino)-terphenyl, 4-(*N,N*-diphenylamino)biphenyl-4´-boronic acid,[2] $Ph_2P(C_6H_4)_2NPh_2$ (**L2$_1$**),[3] $P[(C_6H_4)_2NPh_2]_3$ (**L2$_3$**),[4] $P[(C_6H_4)_3NPh_2]_3$ (**L3$_3$**),[4] (4´´-(*N,N*-diphenylamino)terphenyl-4-yl)triphenylphosphonium bromide (**3$_1$[Br]**)[5] were synthesized according to the published procedures. Tetrahydrofuran (THF), toluene and diethyl ether were distilled over Na-benzophenone ketyl under a nitrogen atmosphere prior to use. Other reagents and solvents were used as received. The solution ^1H, $^{31}P\{^1H\}$, $^{13}C\{^1H\}$ and ^1H–^1H COSY NMR spectra were recorded on Bruker Avance 400, AMX-400 and JEOL ECZ500R/M3 spectrometers. Mass spectra were recorded on a Bruker maXis II ESI-QTOF instrument in the ESI$^+$ mode. Microanalyses were carried out at the analytical laboratory of the University of Eastern Finland.

Syntheses

$Ph_2P(C_6H_4)_3NPh_2$ (**L3$_1$**). A solution of 4-bromo-4´´-(*N,N*-diphenylamino)-terphenyl (2.35 g, 5.0 mmol) in freshly distilled THF (80 ml) was cooled to –78 ºC, and a 1.6 M solution of *n*-BuLi in hexane (3.4 mL, 5.5 mmol) was added dropwise within 15 min. to give a light yellow clear solution, which was stirred at this temperature for 30 min. Then solution was treated dropwise with neat PPh_2Cl (1.10 g, 5.0 mmol). The reaction mixture was allowed to reach room temperature and was stirred for additional 1 h. Then solution was quenched with methanol (5 ml) and solvent was evaporated. The solid residue was washed with methanol (3 × 25 ml) and dried. Crude **L3$_1$** was purified by column chromatography (Silica gel 70–230 mesh, 3×20 cm, eluent dichloromethane-hexane, 1:1 v/v). The solvents were evaporated to afford white precipitate (1.74 g, 60 %). $^{31}P\{^1H\}$ NMR (CD_2Cl_2; 298 K; δ): –5.6 (s, 1P, PPh_2). ^1H NMR (CD_2Cl_2, 298 K; *δ*): 7.71 (br s, 4H, $(C_6H_4)_3$), 7.65–7.69 (m, 2H, $(C_6H_4)_3$), 7.38–7.46 (m, 12H, $(C_6H_4)_3$ + *ortho, meta, para*-H PPh_2), 7.27–7.36 (m, 4H, *meta*-H NPh_2), 7.15–7.18 (m, 6H, $(C_6H_4)_3$ + *ortho*-H NPh_2), 7.09 (t, 2H, J_{HH} 6.9 Hz, *para*-H NPh_2). $^{13}C\{^1H\}$ NMR (CD_2Cl_2; 298 K; δ): 148.2, 148.0, 141.5, 140.4, 139.3, 137.9 (d, J = 11.3 Hz), 136.9 (d, J = 11.4 Hz), 134.9, 134.8, 134.7, 134.3 (d, *J* = 19.7 Hz), 129.9, 129.4, 129.1 (d, *J* = 6.9 Hz), 128.1, 127.9, 127.5, 127.4, 125.1, 124.3, 123.7. Anal. Calc. for $C_{42}H_{32}NP$: C, 86.72; H, 5.55; N, 2.41. Found: C 86.90; H 5.43; N, 2.33.

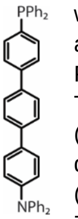

General procedure for the preparation of 2$_2$–2$_4$[Br], **3$_2$[Br]**, **1$_1$2$_1$[Br]**, **1$_1$3$_1$[B]** and **2$_1$3$_1$[Br]**. *N,N*-diphenylamino-bromoaryl or phenylbromide (for **2$_3$[Br]**) (1.00 eq.), $NiBr_2$ (0.20 eq.), appropriate phosphine (1.05 eq.) and ethylene glycol (3–5 mL) were placed in a 15 mL sealed tube, degassed and placed under a nitrogen atmosphere. The suspension was stirred for 5–48 hours at 180–200 ºC until the solution became homogeneous. Then the reaction mixture was cooled down to room temperature and poured into dichloromethane (50 mL). The organic layer was washed with water (3 × 100 mL), dried over anhydrous Na_2SO_4 for 30 min, filtered through a pad of Celite and evaporated *in vacuo*. The residue was purified by column chromatography (Silica gel 70-230 mesh, ⌀3×20 cm, eluent dichloromethane-methanol, 99:1→92:8 v/v mixture) to afford phosphonium salts as amorphous solids.

2$_2$[Br]. Prepared from 4-bromo-4´-(*N,N*-diphenylamino)-biphenyl (0.20 g, 0.50 mmol), $NiBr_2$ (0.03 g, 0.10 mmol) and **L2$_1$** (0.26 g, 0.53 mmol) for 12 h at 180 ºC to afford greenish solid (0.31 g, 77 %). ESI-MS (*m/z*): [M]$^+$ 825.3406 (calcd 825.3399). ^1H NMR (CD_2Cl_2, 298 K; *δ*): 7.95–7.99 (m, *para*-H PPh_2^+ and -biph-, 6H), 7.83 (td, J_{HH} = 7.8, 3.6 Hz, *meta*-H PPh_2^+, 4H), 7.68–7.76 (m, *ortho*-H PPh_2^+ and -biph-, 8H), 7.61 (d, J_{HH} = 8.7 Hz, -biph-, 4H), 7.34 (dd, J_{HH} = 7.9 Hz, *meta*-H NPh_2, 8H), 7.09–7.19 (m, *para*+*ortho*-H NPh_2 and -biph-, 16H). $^{31}P\{^1H\}$ NMR (CD_2Cl_2; 298 K; δ): 23.2 (s, 1P, PAr_4^+). $^{13}C\{^1H\}$ NMR (CD_2Cl_2; 298 K; δ): 149.9, 148.3, 147.6, 136.2, 135.5 (d, *J* = 10.7 Hz), 135.0 (d, *J* = 10.3 Hz), 131.2 (d, *J* = 12.9 Hz), 131.2, 130.1. 128.7, 128.5, 125.8, 124.5, 123.0, 118.8 (d, *J* = 89.9 Hz), 115.0 (d, J = 92.6 Hz). Anal. Calcd for $C_{60}H_{46}BrN_2P$: C, 79.55; H, 5.12; N, 3.09. Found: C, 79.28; H, 4.99; N, 2.87.

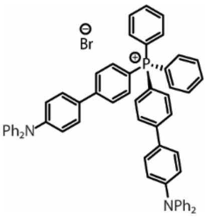

2$_3$[Br]. Prepared from phenylbromide (0.10 g, 0.64 mmol), $NiBr_2$ (0.03 g, 0.13 mmol) and **L2$_3$** (0.66 g, 0.67 mmol) in 5 h at 180 ºC to afford greenish solid (0.59 g, 80 %). ESI-MS (*m/z*): [M]$^+$ 1068.4422 (calcd 1068.4447). ^1H NMR (CD_2Cl_2, 298 K; *δ*): 7.96–7.99 (m, *para*-H PPh^+ and -biph-, 7H), 7.84 (td, J_{HH} = 7.7, 3.7 Hz, *meta*-H PPh^+, 2H), 7.71–7.79 (m, *ortho*-H PPh^+ and -biph-, 8H), 7.62 (d, J_{HH} = 8.7 Hz, -biph-, 6H), 7.34 (dd, J_{HH} = 7.9 Hz, *meta*-H NPh_2, 12H), 7.12–7.19 (m, *para*+*ortho*-H NPh_2 and -biph-, 24H). $^{31}P\{^1H\}$ NMR (CD_2Cl_2; 298 K; δ): 22.9 (s, 1P, PAr_4^+). $^{13}C\{^1H\}$ NMR (CD_2Cl_2; 298 K; δ): 149.9, 148.3, 147.6, 136.2, 135.5 (d, *J* = 10.7 Hz), 135.0 (d, *J* = 10.4 Hz), 131.2, 131.1, 130.1. 128.7, 128.5, 125.8, 124.5, 123.0, 119.0 (d, *J* = 90.1 Hz), 115.4 (d, J = 92.7 Hz). Anal. Calcd for $C_{78}H_{59}BrN_3P$: C, 81.52; H, 5.17; N, 3.66. Found: C, 81.75; H, 5.00; N, 3.49.

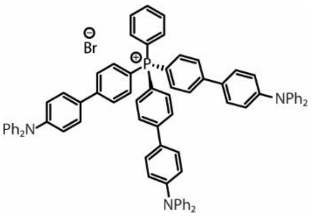

2_4[Br]. Prepared from 4-bromo-4´-(*N,N*-diphenylamino)-biphenyl (0.20 g, 0.50 mmol), $NiBr_2$ (0.02 g, 0.10 mmol) and **$L2_3$** (0.53 g, 0.53 mmol) for 48 h at 180 ºC to afford green solid (0.25 g, 35 %). ESI-MS (*m/z*): $[M]^+$ 1312.5511 (calcd 1312.5527). ^1H NMR (CD_2Cl_2, 298 K; *δ*): 7.98 (dd, J_{HH} = 8.4, 3.0 Hz, -biph-, 6H), 7.76 (dd, J_{HH} = 12.6, 8.4 Hz, biph-, 8H), 7.63 (d, J_{HH} = 8.7 Hz, -biph-, 8H), 7.35 (dd, J_{HH} = 7.8 Hz, *meta*-H NPh_2, 16H), 7.12–7.19 (m, *para+ortho*-H NPh_2 and -biph-, 32H). $^{31}P\{^1H\}$ NMR (CD_2Cl_2; 298 K; δ): 22.6 (s, 1P, PAr_4^+). $^{13}C\{^1H\}$ NMR (CD_2Cl_2; 298 K; δ): 149.9, 148.2, 147.6, 135.5 (d, *J* = 10.8 Hz), 131.3, 130.1. 128.7, 128.5, 125.8, 124.5, 123.0, 115.7 (d, J = 92.9 Hz). Anal. Calcd for $C_{96}H_{72}BrN_4P$: C, 82.80; H, 5.21; N, 4.02. Found: C, 82.97; H, 5.01; N, 4.11.

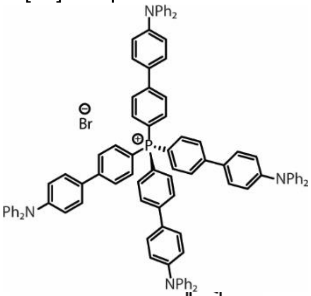

3_2[Br]. Prepared from 4-bromo-4´´-(*N,N*-diphenylamino)-terphenyl (0.35 g, 0.74 mmol), $NiBr_2$ (0.03 g, 0.02 mmol) and **$L3_1$** (0.45 g, 0.77 mmol) in 24 h at 200 ºC to afford yellowish precipitate (0.27 g, 34 %). ESI-MS (*m/z*): $[M]^+$ 977.3973 (calcd 977.4025). ^1H NMR (CD_2Cl_2, 298 K; *δ*): 8.03–8.06 (m,-terph-, 4H), 7.92–7.96 (m, *para*-H PPh_2^+, 2H), 7.70–7.87 (m, *ortho+meta*-H PPh_2^+ and -terph-, 20H), 7.52 (d, J_{HH} = 8.6 Hz, -terph-, 4H), 7.28 (dd, J_{HH} = 7.6 Hz, *meta*-H NPh_2, 8H), 7.14–7.16 (m, *ortho*-H NPh_2 and -terph-, 12H), 7.05 (t, J_{HH} = 7.3 Hz, *para*-H NPh_2, 4H). $^{31}P\{^1H\}$ NMR (CD_2Cl_2; 298 K; δ): 23.3 (s, 1P, PAr_4^+). $^{13}C\{^1H\}$ NMR (CD_2Cl_2; 298 K; δ): 148.5, 148.1, 142.2, 136.9, 136.3 (d, *J* = 2.7 Hz), 135.6 (d, *J* = 10.7 Hz), 135.1 (d, *J* = 10.4 Hz), 133.3 (d, *J* = 12.9 Hz), 129.9, 129.3 (d, *J* = 13.3 Hz), 128.5, 128.0 (d, *J* = 37.1 Hz), 125.2, 124.0, 123.9, 118.5 (d, *J* = 89.9 Hz), 116.1 (d, *J* = 91.9 Hz). Anal. Calcd for $C_{72}H_{54}BrN_2P$: C, 81.73; H, 5.14; N, 2.65. Found: C, 81.51; H, 5.34; N, 2.79.

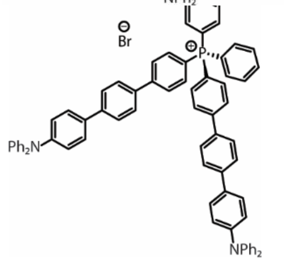

1_12_1[Br]. Prepared from 4-bromo-(*N,N*-diphenyl)-aniline (0.08 g, 0.25 mmol), $NiBr_2$ (0.01 g, 0.05 mmol) and **$L2_1$** (0.13 g, 0.26 mmol) in 6 h at 180 ºC to afford greenish precipitate (0.10 g, 45 %). ESI-MS (*m/z*): $[M]^+$ 749.3069 (calcd 749.3086). ^1H NMR (CD_2Cl_2, 298 K; *δ*): 7.86–7.90 (m, *para*-H PPh_2^+ and -biph-, 4H), 7.74 (td, J_{HH} = 7.9, 3.6 Hz, *meta*-H PPh_2^+, 4H), 7.60–7.67 (m, *ortho*-H PPh_2^+, -ph- and -biph-, 6H), 7.54 (d, J_{HH} = 8.7 Hz, -biph-, 2H), 7.34 (m, *meta*-H NPh_2, 4H), 7.23–7.41 (m, *ortho*-H NPh_2, -ph- and -biph-, 12H), 7.04–7.13 (m, *para*-H NPh_2 and -ph-, 10H). $^{31}P\{^1H\}$ NMR ($CDCl_3$; 298 K; δ): 22.3 (s, 1P, PAr_4^+). $^{13}C\{^1H\}$ NMR (CD_2Cl_2; 298 K; δ): 149.2, 147.4 (d, *J* = 3.1 Hz), 147.1, 144.9, 135.5 (d, *J* = 11.7 Hz), 135.3 (d, *J* = 2.9 Hz), 134.8 (d, *J* = 10.6 Hz), 134.3 (d, *J* = 10.3 Hz), 130.8, 130.5, 130.4, 130.1, 129.5, 128.1, 128.0, 126.3, 127.0, 126.4, 125.2, 123.9, 122.5, 119.4, 118.7, 118.6, 115.9, 115.1, 103.3 (d, *J* = 99.8 Hz). Anal. Calcd for $C_{54}H_{42}BrN_2P$: C, 78.16; H, 5.10; N, 3.38. Found: C, 78.50; H, 4.92; N, 3.23.

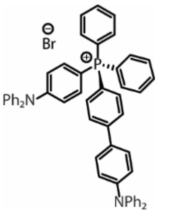

2_13_1[Br]. Prepared from 4-bromo-4´-(*N,N*-diphenylamino)-biphenyl (0.12 g, 0.30 mmol), $NiBr_2$ (0.02 g, 0.06 mmol) and **$L3_1$** (0.18 g, 0.32 mmol) in 48 h at 200 ºC to afford yellow precipitate (0.12 g, 40 %). ESI-MS (*m/z*): $[M]^+$ 901.3642 (calcd 901.3712). ^1H NMR ($CDCl_3$, 298 K; *δ*): 8.05–8.07 (m, -terph-, 2H), 7.96–8.00 (m, *para*-H PPh_2^+ and -biph-, 4H), 7.69–7.86 (m, *meta+ortho*-H PPh_2^+, -terph-, -biph-, 16H), 7.58–7.63 (m, -terph- and -biph-, 4H), 7.30–7.37 (m, *meta*-H NPh_2, 8H), 7.07–7.19 (m, *ortho+para*-H NPh_2 and -biph-, -terph-, 16H). $^{31}P\{^1H\}$ NMR (CD_2Cl_2; 298 K; δ): 23.4 (s, 1P, PAr_4^+). $^{13}C\{^1H\}$ NMR (CD_2Cl_2; 298 K; δ): 149.9, 148.5, 148.4, 148.1, 147.6, 142.2, 136.9, 136.3, 135.6 (d, *J* = 4.8 Hz), 135.5 (d, *J* = 4.9 Hz), 135.0 (d, *J* = 10.4 Hz), 133.9, 131.3, 131.2, 130.1, 129.9, 129.3, 129.2, 128.7, 128.6, 128.4, 128.2, 127.8, 125.8, 125.2, 124.5, 124.0, 123.9, 123.0, 118.6 (d, *J* = 89.9 Hz), 116.2 (d, *J* = 91.9 Hz), 114.9 (d, *J* = 92.6 Hz). Anal. Calcd for $C_{66}H_{50}BrN_2P$: C, 80.72; H, 5.13; N, 2.85. Found: C, 80.55; H, 5.33; N, 2.87.

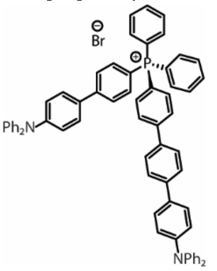

1_13_1[Br]. Prepared from 4-bromo-(*N,N*-diphenyl)-aniline (0.15 g, 0.46 mmol), $NiBr_2$ (0.02 g, 0.09 mmol) and **$L3_1$** (0.28 g, 0.48 mmol) in 24 h at 200 ºC to afford yellow precipitate (0.23 g, 52 %). ESI-MS (*m/z*): $[M]^+$ 825.3375 (calcd 825.3399). ^1H NMR (CD_2Cl_2, 298 K; *δ*): 7.97–7.99 (m, -terph-, 2H), 7.88–7.91 (m, *para*-H PPh_2^+, 2H), 7.64–7.76 (m, *meta+ortho*-H PPh_2^+, -ph- and -terph-, 14H), 7.54 (d, J_{HH} = 8.4 Hz, -terph-, 2H), 7.34 (dd, *J* = 7.7 Hz, *meta*-H NPh_2, 4H), 7.23–7.42 (m, *ortho*-H NPh_2, -ph- and -terph-, 12H), 7.03–7.12 (m, *para*-H NPh_2 and -ph-, 10H). $^{31}P\{^1H\}$ NMR (CD_2Cl_2; 298 K; δ): 22.4 (s, 1P, PAr_4^+). $^{13}C\{^1H\}$ NMR (CD_2Cl_2; 298 K; δ): 147.9, 147.6, 144.9, 141.5, 136.5, 135.6 (d, *J* = 11.7 Hz), 135.4 (d, *J* = 3.1 Hz), 134.9 (d, *J* = 10.6 Hz), 134.3 (d, *J* = 10.6 Hz), 133.4, 130.5 (d, *J* = 12.8 Hz), 130.2, 129.4, 128.6 (d, *J* = 13.1 Hz), 127.9, 127.7, 127.3, 127.0, 126.5, 124.6, 123.5, 123.3, 119.3, 118.7, 118.6. Anal. Calcd for $C_{60}H_{46}BrN_2P$: C, 79.55; H, 5.12; N, 3.09. Found: C, 80.00; H, 5.33; N, 3.01.

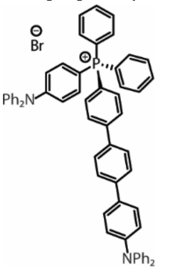

General procedure for the preparation of 2_2–2_4[OTf], **3_1[OTf]**, **3_2[OTf]**, **1_12_1[OTf]**, **1_13_1[OTf]** and **2_13_1[OTf]**. A solution of silver(I) trifluoromethanesulfonate (1.05 eq.) in acetone (2 mL) was added to a solution of ***Y_n*[Br]** (1.00 eq.) in dichlorometane (10 mL). The reaction mixture was stirred at room temperature for 20 min., and the resulting suspension was filtered through a Celite pad and evaporated *in vacuo.* The residue was purified by column chromatography (Silica gel 70-230 mesh, ⌀3×10 cm, eluent dichloromethane-methanol, 95:5 v/v mixture) to afford ***Y_n*[OTf]**.

2_2[OTf]. Prepared from **2_2[Br]** (0.10 g, 0.11 mmol) and AgOTf (0.03 g, 0.12 mmol) to afford greenish precipitate (0.10 g, 96 %). ESI-MS (*m/z*): $[M]^+$ 825.3386 (calcd 825.3399). ^{1}H NMR (CD_2Cl_2, 298 K; *δ*): 7.94–7.97 (m, *para*-H PPh_2^+ and -biph-, 6H), 7.81 (td, J_{HH} = 7.9, 3.6 Hz, *meta*-H PPh_2^+, 4H), 7.66–7.74 (m, *ortho*-H PPh_2^+ and -biph-, 8H), 7.60 (d, J_{HH} = 8.7 Hz, -biph-, 4H), 7.34 (dd, J_{HH} = 7.9 Hz, *meta*-H NPh_2, 8H), 7.12–7.19 (m, *para*+*ortho*-H NPh_2 and -biph-, 16H). ^{31}P{^{1}H} NMR (CD_2Cl_2; 298 K; δ): 23.3 (s, 1P, PAr_4^+). ^{13}C{^{1}H} NMR (CD_2Cl_2; 298 K; δ): 149.9, 148.3 (d, *J* = 3.0 Hz), 147.6, 136.2 (d, J = 2.8 Hz), 135.5 (d, *J* = 10.7 Hz), 135.0 (d, *J* = 10.4 Hz), 131.1 (d, *J* = 12.8 Hz), 131.2, 130.1. 128.7, 128.5, 125.8, 124.5, 123.0, 118.8 (d, *J* = 90.0 Hz), 115.0 (d, J = 92.6 Hz). Anal. Calcd for $C_{61}H_{46}F_3N_2O_3PS$: C, 75.14; H, 4.76; N, 2.87. Found: C, 74.97; H, 4.71; N, 2.90.

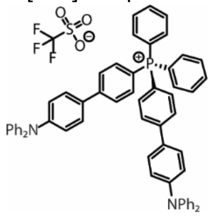

2_3[OTf]. Prepared from **2_3[Br]** (0.15 g, 0.13 mmol) and AgOTf (0.04 g, 0.14 mmol) to afford green precipitate (0.16 g, 98 %). ESI-MS (*m/z*): $[M]^+$ 1068.4416 (calcd 1068.4447). ^{1}H NMR (CD_2Cl_2, 298 K; *δ*): 7.95–7.98 (m, *para*-H PPh^+ and -biph-, 7H), 7.84 (td, J_{HH} = 7.7, 3.7 Hz, *meta*-H PPh^+, 2H), 7.71–7.79 (m, *ortho*-H PPh^+ and -biph-, 8H), 7.62 (d, J_{HH} = 8.8 Hz, -biph-, 6H), 7.34 (dd, J_{HH} = 7.9 Hz, *meta*-H NPh_2, 12H), 7.12–7.19 (m, *para*+*ortho*-H NPh_2 and -biph-, 24H). ^{31}P{^{1}H} NMR (CD_2Cl_2; 298 K; δ): 23.0 (s, 1P, PAr_4^+). ^{13}C{^{1}H} NMR (CD_2Cl_2; 298 K; δ): 149.9, 148.3 (d, *J* = 3.0 Hz), 147.6, 136.1, 135.5 (d, *J* = 10.7 Hz), 135.0 (d, *J* = 10.3 Hz), 131.3, 131.1 (d, *J* = 12.9 Hz), 130.1, 128.7, 128.5, 125.8, 124.5, 123.0, 119.0 (d, *J* = 90.0 Hz), 115.4 (d, *J* = 92.7 Hz). Anal. Calcd for $C_{79}H_{59}F_3N_3O_3PS$: C, 77.88; H, 4.88; N, 3.45. Found: C, 78.02; H, 4.91; N, 3.33.

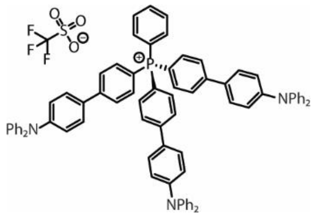

2_4[OTf]. Prepared from **2_4[Br]** (0.10 g, 0.07 mmol) and AgOTf (0.02 g, 0.08 mmol) to afford green precipitate (0.10 g, 93 %). ESI-MS (*m/z*): $[M]^+$ 1312.5491 (calcd 1312.5527). ^{1}H NMR (CD_2Cl_2, 298 K; *δ*): 7.97 (dd, $J_{HH, PH}$ = 8.7, 3.2 Hz, -biph-, 6H), 7.76 (dd, $J_{HH, PH}$ = 12.6, 8.7 Hz, biph-, 8H), 7.62 (d, J_{HH} = 8.9 Hz, -biph-, 8H), 7.35 (dd, J_{HH} = 7.8 Hz, *meta*-H NPh_2, 16H), 7.10–7.20 (m, *para*+*ortho*-H NPh_2 and -biph-, 32H). ^{31}P{^{1}H} NMR (CD_2Cl_2; 298 K; δ): 22.6 (s, 1P, PAr_4^+). ^{13}C{^{1}H} NMR (CD_2Cl_2; 298 K; δ): 149.9, 148.2, 147.7, 135.4 (d, *J* = 10.7 Hz), 131.3, 130.1. 128.7, 128.5, 125.8, 124.5, 123.0, 115.7 (d, J = 92.9 Hz). Anal. Calcd for $C_{97}H_{72}F_3N_4O_3PS$: C, 79.71; H, 4.97; N, 3.83. Found: C, 79.61; H, 5.11; N, 3.78.

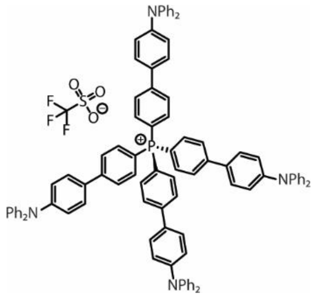

3_1[OTf]. Prepared from **3_1[Br]** (0.10 g, 0.14 mmol) and AgOTf (0.04 g, 0.14 mmol) to afford yellow precipitate (0.10 g, 89 %). ESI-MS (*m/z*): $[M]^+$ 658.2671 (calcd 658.2664). ^{1}H NMR (CD_2Cl_2, 298 K; *δ*): 8.02–8.05 (m,-terph-, 2H), 7.95–7.99 (m, *para*-H PPh_3^+, 3H), 7.68–7.84 (m, *ortho*+*meta*-H PPh_3^+ and -terph-, 18H), 7.59 (d, J_{HH} = 8.8 Hz, -terph-, 2H), 7.32 (dd, J_{HH} = 7.6 Hz, *meta*-H NPh_2, 4H), 7.12–7.18 (m, *ortho*-H NPh_2 and -terph-, 6H), 7.10 (t, J_{HH} = 7.6 Hz, *para*-H NPh_2, 2H). ^{31}P{^{1}H} NMR (CD_2Cl_2; 298 K; δ): 23.7 (s, 1P, PAr_4^+). ^{13}C{^{1}H} NMR (CD_2Cl_2; 298 K; δ): 148.5, 148.1, 142.2, 136.9, 135.3, 135.6 (d, *J* = 10.7 Hz), 135.0 (d, *J* = 10.3 Hz), 133.9, 131.2 (d, *J* = 12.9 Hz), 130.0, 129.3 (d, *J* = 13.3 Hz), 128.3 (d, *J* = 19.3 Hz), 125.3, 124.1, 123.9, 118.3 (d, *J* = 89.8 Hz), 115.9 (d, *J* = 91.8 Hz). Anal. Calcd for $C_{49}H_{37}F_3NO_3PS$: C, 72.85; H, 4.62; N, 1.73. Found: C, 73.02; H, 4.54; N, 1.61.

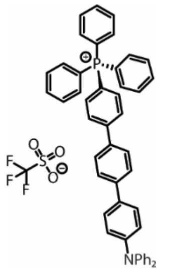

3_2[OTf]. Prepared from **3_2[Br]** (0.12 g, 0.11 mmol) and AgOTf (0.03 g, 0.12 mmol) to afford yellow precipitate (0.11 g, 92 %). ESI-MS (*m/z*): $[M]^+$ 977.3976 (calcd 977.4025). ^{1}H NMR (CD_2Cl_2, 298 K; *δ*): 8.04–8.07 (m,-terph-, 4H), 7.96–8.01 (m, *para*-H PPh_2^+, 2H), 7.72–7.86 (m, *ortho*+*meta*-H PPh_2^+ and -terph-, 20H), 7.59 (d, J_{HH} = 8.7 Hz, -terph-, 4H), 7.33 (dd, J_{HH} = 7.6 Hz, *meta*-H NPh_2, 8H), 7.15–7.18 (m, *ortho*-H NPh_2 and -terph-, 12H), 7.10 (t, J_{HH} = 7.3 Hz, *para*-H NPh_2, 4H). ^{31}P{^{1}H} NMR (CD_2Cl_2; 298 K; δ): 23.5 (s, 1P, PAr_4^+). ^{13}C{^{1}H} NMR (CD_2Cl_2; 298 K; δ): 148.5, 148.1, 142.2, 136.9, 136.3 br s, 135.6 (d, *J* = 10.7 Hz), 135.0 (d, *J* = 10.4 Hz), 133.9, 133.2 (d, *J* = 12.9 Hz), 129.9, 129.3 (d, *J* = 13.3 Hz), 128.4, 128.2, 127.9, 125.3, 124.0, 123.9, 118.5 (d, *J* = 90.0 Hz), 116.0 (d, *J* = 91.9 Hz). Anal. Calcd for $C_{73}H_{54}F_3N_2O_3PS$: C, 77.78; H, 4.83; N, 2.49. Found: C, 77.39; H, 4.92; N, 2.31.

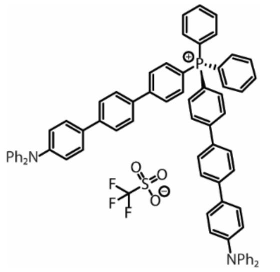

1_12_1[OTf]. Prepared from **1_12_1[Br]** (0.09 g, 0.11 mmol) and AgOTf (0.03 g, 0.11 mmol) to afford greenish precipitate (0.08 g, 93 %). ESI-MS (*m/z*): $[M]^+$ 749.3073 (calcd 749.3086). ^{1}H NMR (CD_2Cl_2, 298 K; *δ*): 7.85–7.89 (m, *para*-H PPh_2^+ and -biph-, 4H), 7.73 (td, J_{HH} = 7.9, 3.6 Hz, *meta*-H PPh_2^+, 4H), 7.59–7.66 (m, *ortho*-H PPh_2^+, -ph- and -biph-, 6H), 7.54 (d, J_{HH} = 8.7 Hz, -biph-, 2H), 7.34 (m, *meta*-H NPh_2, 4H), 7.23–7.31 (m, *ortho*-H NPh_2, -ph- and -biph-, 12H), 7.04–7.14 (m, *para*-H NPh_2 and -ph-, 10H). ^{31}P{^{1}H} NMR (CD_2Cl_2; 298 K; δ): 22.3 (s, 1P, PAr_4^+). ^{13}C{^{1}H} NMR (CD_2Cl_2; 298 K; δ): 149.3, 147.4 (d, *J* = 3.5 Hz), 147.1, 145.0, 135.5 (d, *J* = 11.7 Hz), 135.3 (d, *J* = 3.0 Hz), 134.8 (d, *J* = 10.7 Hz), 134.3 (d, *J* = 10.3 Hz), 130.9, 130.5, 130.4, 130.1, 129.5, 128.1, 128.0, 127.8, 127.0, 126.4, 125.3, 124.0, 122.5, 119.4, 118.6 (d, *J* = 13.8 Hz), 115.5 (d, *J* = 93.0 Hz), 103.3 (d, *J* = 99.6 Hz). Anal. Calcd for $C_{55}H_{42}F_3N_2O_3PS$: C, 73.48; H, 4.71; N, 3.12. Found: C, 73.22; H, 4.89; N, 3.01.

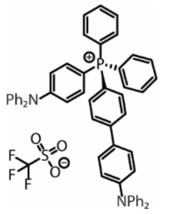

2$_1$3$_1$[OTf]. Prepared from **2$_1$3$_1$[Br]** (0.15 g, 0.15 mmol) and AgOTf (0.04 g, 0.16 mmol) to afford yellow precipitate (0.14 g, 90 %). ESI-MS (*m/z*): $[M]^+$ 901.3656 (calcd 901.3712). ^{1}H NMR ($CDCl_3$, 298 K; *δ*): 8.03–8.06 (m, -terph-, 2H), 7.95–8.00 (m, *para*-H PPh_2^+ and -biph-, 4H), 7.68–7.85 (m, *meta+ortho*-H PPh_2^+, -terph-, -biph-, 16H), 7.58–7.62 (m, -terph- and -biph-, 4H), 7.30–7.37 (m, *meta*-H NPh_2, 8H), 7.00–7.19 (m, *ortho+para*-H NPh_2 and -biph-, -terph-, 16H). ^{31}P{^{1}H} NMR (CD_2Cl_2; 298 K; δ): 23.4 (s, 1P, PAr_4^+). ^{13}C{^{1}H} NMR (CD_2Cl_2; 298 K; δ): 149.9, 148.5, 148.4, 148.1, 147.6, 142.2, 136.9, 136.3 (d, *J* = 2.6 Hz), 135.6 (d, *J* = 4.6 Hz), 135.5 (d, *J* = 4.6 Hz), 135.0 (d, *J* = 10.4 Hz), 133.9, 131.3, 131.1, 130.1, 129.9, 129.3, 129.2, 128.7, 128.6, 128.4, 128.2, 127.8, 125.8, 125.2, 124.5, 124.0, 123.9, 123.0, 118.6 (d, *J* = 90.0 Hz), 116.2 (d, *J* = 91.9 Hz), 114.9 (d, *J* = 92.6 Hz). Anal. Calcd for $C_{67}H_{50}F_3N_2O_3PS$: C, 76.56; H, 4.79; N, 2.67. Found: C, 76.90; H, 4.41; N, 2.58.

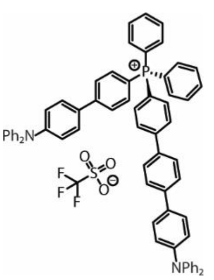

1$_1$3$_1$[OTf]. Prepared from **1$_1$3$_1$[Br]** (0.10 g, 0.11 mmol) and AgOTf (0.03 g, 0.12 mmol) to afford yellow precipitate (0.10 g, 93 %). ESI-MS (*m/z*): $[M]^+$ 825.3415 (calcd 825.3399). ^{1}H NMR (CD_2Cl_2, 298 K; *δ*): 7.96–7.98 (m, -terph-, 2H), 7.88 (t, *J* = 7.2 Hz, *para*-H PPh_2^+, 2H), 7.64–7.76 (m, *meta+ortho*-H PPh_2^+, -ph- and -terph-, 14H), 7.54 (d, J_{HH} = 8.6 Hz, -terph-, 2H), 7.34 (dd, *J* = 7.8 Hz, *meta*-H NPh_2, 4H), 7.24–7.32 (m, *ortho*-H NPh_2, -ph- and -terph-, 12H), 7.04–7.13 (m, *para*-H NPh_2 and -ph-, 10H). ^{31}P{^{1}H} NMR (CD_2Cl_2; 298 K; δ): 22.4 (s, 1P, PAr_4^+). ^{13}C{^{1}H} NMR (CD_2Cl_2; 298 K; δ): 147.9, 147.6, 145.0, 141.5, 136.5, 135.6 (d, *J* = 11.8 Hz), 135.4 (d, *J* = 3.0 Hz), 134.9 (d, *J* = 10.7 Hz), 134.3 (d, *J* = 10.3 Hz), 133.5, 130.5 (d, *J* = 12.8 Hz), 130.2, 129.4, 128.5 (d, *J* = 13.2 Hz), 127.9, 127.7, 127.3, 127.0, 126.5, 124.7, 123.5, 123.3, 119.3, 118.7, 118.6. Anal. Calcd for $C_{61}H_{46}F_3N_2O_3PS$: C, 75.14; H, 4.76; N, 2.87. Found: C, 74.98; H, 4.80; N, 2.88.

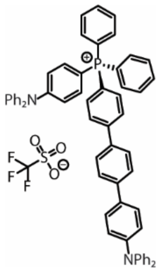

General procedure for the preparation of 2$_1$Me[OTf], 2$_3$Me[OTf] and **3$_3$Me[OTf]**. A degassed solution of phosphine **L2$_1$**, **L2$_3$** or **L3$_3$** (1.00 eq.) in dichloromethane (10 mL) was cooled to 0 ºC, and excess of methyl trifluoromethanesulfonate (2.00 eq.) was added in one portion. The reaction mixture was stirred at this temperature for 20 min., and then was allowed to reach room temperature. To resulting clear solution was added hexanes (40 ml). The amorphous greenish residue was washed with diethyl ether (3 × 20 ml), pentane (3 × 20 ml), and dried *in vacuo*. The residue was purified by column chromatography (Silica gel 70-230 mesh, ⌀3×20 cm, eluent dichloromethane-methanol, 98:2→95:5 v/v mixture) to afford ***Y$_n$*Me[OTf]**.

2$_1$Me[OTf]. Prepared from **L2$_1$** (0.25 g, 0.50 mmol) and methyl trifluoromethanesulfonate (0.17 g, 1.00 mmol) to afford greenish precipitate (0.31 g, 93 %). ESI-MS (*m/z*): $[M]^+$ 520.2140 (calcd 520.2194). ^{1}H NMR (CD_2Cl_2, 298 K; *δ*): 7.87–7.93 (m, *para*-H PPh_2^+ and -biph-, 4H), 7.66–7.78 (m, *meta+ortho*-H PPh_2^+ and -biph-, 10H), 7.58 (d, J_{HH} = 8.8 Hz, -biph-, 4H), 7.34 (dd, J_{HH} = 7.9 Hz, *meta*-H NPh_2, 4H), 7.11–7.18 (m, *para+ortho*-H NPh_2 and -biph-, 8H), 2.89 (d, d, J_{PH} = 13.3 Hz, -Me, 3H). ^{31}P{^{1}H} NMR (CD_2Cl_2; 298 K; δ): 21.5 (s, 1P, $PMeAr_3^+$). ^{13}C{^{1}H} NMR (CD_2Cl_2; 298 K; δ): 149.7, 148.1 (d, *J* = 2.9 Hz), 147.7, 135.9 (d, *J* = 2.9 Hz), 134.1 (d, *J* = 11.1 Hz), 133.6 (d, *J* = 10.7 Hz), 131.5, 131.1 (d, *J* = 12.9 Hz), 130.0. 128.7, 128.5, 125.8, 124.4, 123.1, 119.7 (d, *J* = 89.1 Hz), 116.1 (d, *J* = 91.5 Hz), 10.3 (d, *J* = 58.9 Hz). Anal. Calcd for $C_{38}H_{31}F_3NO_3PS$: C, 68.15; H, 4.67; N, 2.09. Found: C, 67.89; H, 4.88; N, 2.13.

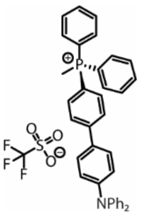

2$_1$Me[Br]. NH_4Br (200 eq.) was added to a solution of **2$_1$Me**[OTf] (1 eq.) in methanol (50 ml) and stirred for 10 min. Then the solvent was removed under vacuum and the phosphonium salt was extracted with dichloromethane (3×5 ml), filtered through Celite pad and evaporated to give greenish residue. The above mentioned procedure was repeated five times, and finally the product was purified by column chromatography (Silica gel 70-230 mesh, ⌀3×10 cm, eluent dichloromethane- methanol, 95:5 v/v mixture) to afford **2$_1$Me**[Br] as a yellowish solid. ^{1}H NMR ($CDCl_3$, 298 K; *δ*): 7.68–7.85 (m, *para+meta+ortho*-H PPh_2^+ and -biph-, 14H), 7.49 (d, J_{HH} = 8.8 Hz, -biph-, 4H), 7.29 (dd, J_{HH} = 8.6 Hz, *meta*-H NPh_2, 4H), 7.07–7.16 (m, *para+ortho*-H NPh_2 and -biph-, 8H), 3.26 (d, d, J_{PH} = 13.2 Hz, -Me, 3H). ^{31}P{^{1}H} NMR ($CDCl_3$; 298 K; δ): 21.7 (s, 1P, $PMeAr_3^+$). ^{31}P{^{1}H} NMR (toluene-d$_8$; 298 K; δ): 22.4 (s, 1P, $PMeAr_3^+$). ^{31}P{^{1}H} NMR (benzene-d$_6$; 298 K; δ): 22.1 (s, 1P, $PMeAr_3^+$). Anal. Calcd for $C_{37}H_{31}BrNP$: C, 74.00; H, 5.20; N, 2.33. Found: C, 73.90; H, 4.95; N, 2.30; S, 0.01.

2$_3$Me[OTf]. Prepared from **L2$_3$** (0.30 g, 0.30 mmol) and methyl trifluoromethanesulfonate (0.10 g, 0.60 mmol) to afford greenish precipitate (0.30 g, 85 %). ESI-MS (*m/z*): $[M]^+$ 1006.4277 (calcd 1006.4290). ^{1}H NMR (CD_2Cl_2, 298 K; *δ*): 7.93 (dd, J_{HH} = 8.4.0, 3.1 Hz, -biph-, 6H), 7.73 (dd, J_{HH} = 13.0, 8.4 Hz, -biph-, 6H), 7.59 (d, J_{HH} = 8.7 Hz, -biph-, 6H), 7.34 (dd, J_{HH} = 7.9 Hz, *meta*-H NPh_2, 12H), 7.11–7.18 (m, *para+ortho*-H NPh_2 and -biph-, 24H), 2.89 (d, d, J_{PH} = 13.3 Hz, -Me, 3H). ^{31}P{^{1}H} NMR (CD_2Cl_2; 298 K; δ): 20.6 (s, 1P, $PMeAr_3^+$). ^{13}C{^{1}H} NMR (CD_2Cl_2; 298 K; δ): 149.8, 148.0 (d, *J* = 2.9 Hz), 147.7, 134.1 (d, *J* = 10.6 Hz), 131.5, 130.0. 128.6, 128.5, 125.8, 124.4, 123.1, 116.6 (d, *J* = 91.8 Hz), 10.6 (d, *J* = 59.5 Hz). Anal. Calcd for $C_{74}H_{57}F_3N_3O_3PS$: C, 76.87; H, 4.97; N, 3.63. Found: C, 77.00; H, 5.05; N, 3.50.

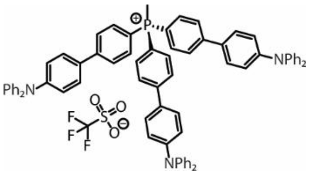

3_3Me[**OTf**]. Prepared from **L3_3** (0.20 g, 0.16 mmol) and methyl trifluoromethanesulfonate (0.06 g, 0.32 mmol) to afford yellow precipitate (0.19 g, 83 %). ESI-MS (*m/z*): $[M]^+$ 1234.5177 (calcd 1234.5229). ^1H NMR (CD_2Cl_2, 298 K; *δ*): 8.02–8.05 (m, -terph-, 6H), 7.76–8.86 (m, -terph-, 18H), 7.59 (d, J_{HH} = 8.7 Hz, -terph-, 6H), 7.32 (dd, J_{HH} = 7.6 Hz, *meta*-H NPh_2, 12H), 7.15–7.18 (m, *ortho*-H NPh_2 and -terph-, 18H), 7.09 (t, J_{HH} = 7.6 Hz, *para*-H NPh_2, 6H), 2.97 (d, d, J_{PH} = 13.3 Hz, -Me, 3H). 2.97 (d, d, J_{PH} = 13.3 Hz, -Me, 3H). $^{31}P\{^1H\}$ NMR (CD_2Cl_2; 298 K; δ): 21.2 (s, 1P, $PMeAr_3^+$). $^{13}C\{^1H\}$ NMR (CD_2Cl_2; 298 K; δ): 148.4, 148.2, 148.1, 142.1, 137.1, 134.2 (d, *J* = 11.1 Hz), 134.0, 129.9, 129.3 (d, *J* = 13.3 Hz), 128.3 (d, *J* = 17.7 Hz), 127.8, 125.3, 124.0, 123.8, 117.5 (d, *J* = 91.0 Hz), 10.5 (d, *J* = 59.3 Hz). Anal. Calcd for $C_{92}H_{69}F_3N_3O_3PS$: C, 79.81; H, 5.02; N, 3.03. Found: C, 80.09; H, 5.04; N, 3.11.

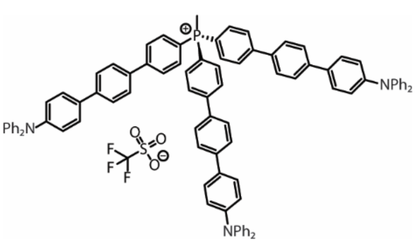

Photophysical measurements

The UV−vis absorption spectra and the emission spectra were performed by a U-3310 spectrophotometer (Hitachi) and a FLS980 fluorometer (Edinburgh Instrument), respectively. The calibration of the excitation and emission wavelength of FLS 980 were carefully done. The samples were prepared in a 1 cm length cuvette with the absorbance of 0.1 at the excitation wavelength (ca. 10^{-6}-10^{-5} M). For the picosecond to sub-nanosecond time-resolved measurements were carried out by a time-correlated single photon counting (TCSPC) system (OB-900L lifetime spectrometer, Edinburgh) with the excitation light source from second harmonic generation (SHG) of 400 nm of pulse-selected tsunami femtosecond laser pulses at 800 nm (Spectra-Physics). The fluorescence was collected at an angle of 90° with respect to the pump beam path. The pump beam passed through a polarizer, which is set at magic angle (54.7°) with respect to the pump polarization, located in front of the detector to eliminate the anisotropy. The temporal resolution was estimated to be 15 ps after removing the instrument broadening.

Computational details

Cations **2_2**$^+$ and **2_13_1**$^+$ were studied with density functional theory (DFT), using hybrid DFT-PBE0 method and hybrid long-range corrected DFT-LRC-ωPBEh method.[6] Gaussian-type, triple-ξ-valence quality basis set with polarization functions (def2-TZVP) was used for all atoms heavier than hydrogen, while a smaller def2-SV(P) basis set was used for hydrogen atoms.[7] Multipole-accelerated resolution-of-the-identity technique was used to speed up the calculations.[8] The geometries of all studied systems were fully optimized within their respective point groups. XYZ coordinates of the optimized structures are included as Supporting Information. No solvation effects were taken into account. The excited states were investigated using the Time-Dependent DFT formalism.[9] The TD-DFT studies were carried out using DFT-LRC-ωPBEh and seminumerical semiJK algorithm was used to speed up the excited state calculations.[10] The $S_0 \rightarrow S_1$ excitation energies were determined at the optimized ground state S_0 geometries and the $S_1 \rightarrow S_0$ emission energies at the optimized S_1 geometries. All electronic structure calculations were carried out with the TURBOMOLE program package (version 7.5).[11]

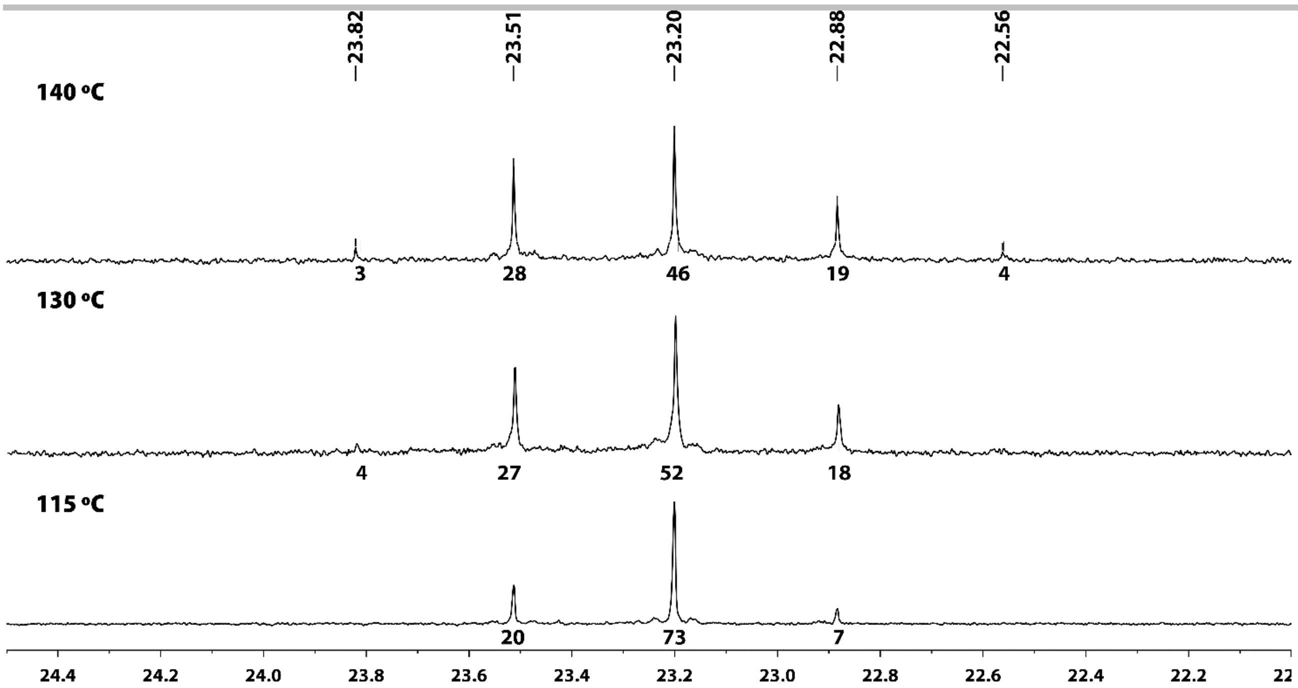

Figure S1. ^{31}P NMR spectra of Pd-catalyzed reactions of $L2_1$ and bromo-*N,N*-diphenyl-aryl amine carried out at three different temperatures (phosphonium ion region, $CDCl_3$, 298 K).

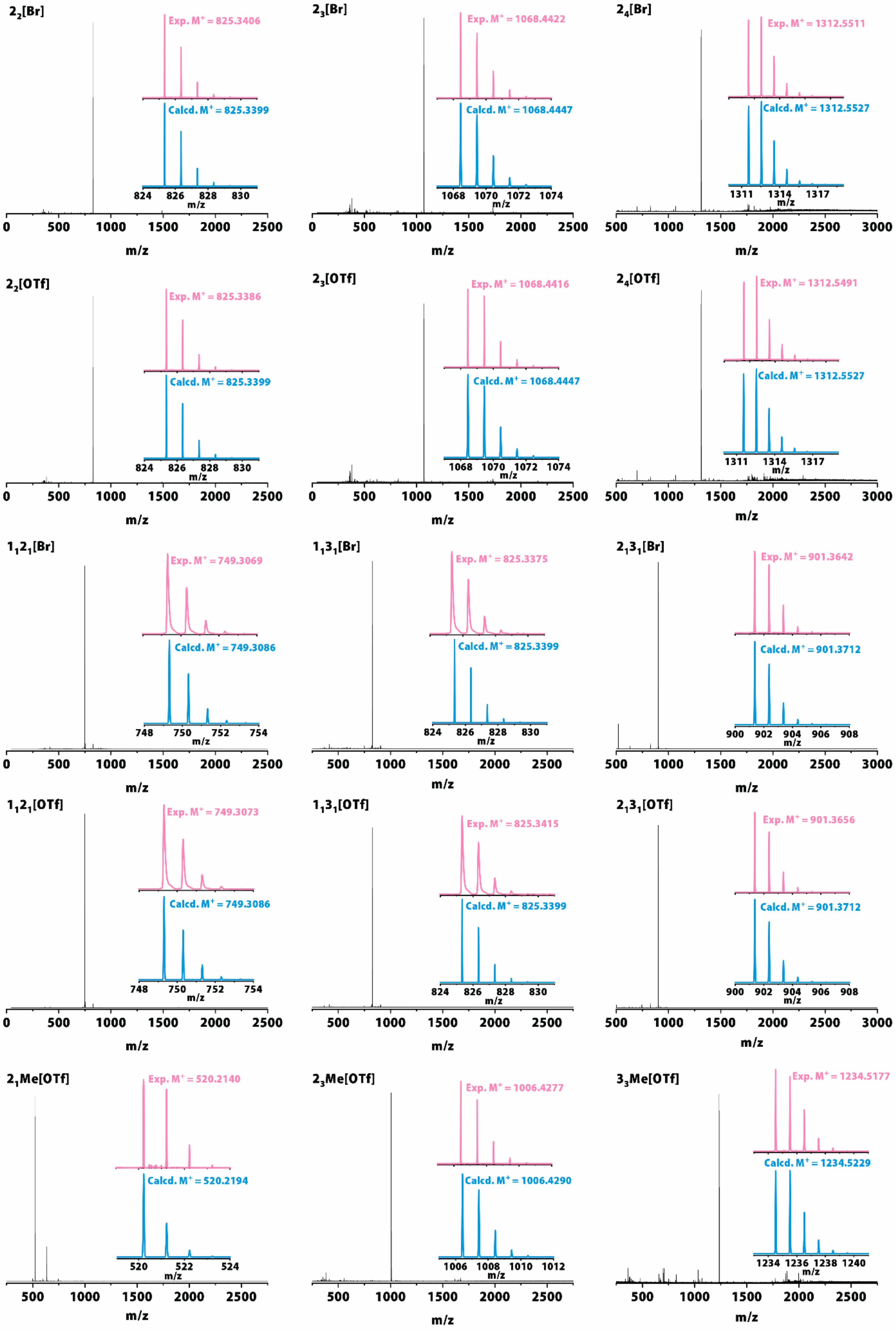
2$_2$[Br]
Exp. M$^+$ = 825.3406
Calcd. M$^+$ = 825.3399
2$_3$[Br]
Exp. M$^+$ = 1068.4422
Calcd. M$^+$ = 1068.4447
2$_4$[Br]
Exp. M$^+$ = 1312.5511
Calcd. M$^+$ = 1312.5527
2$_2$[OTf]
Exp. M$^+$ = 825.3386
Calcd. M$^+$ = 825.3399
2$_3$[OTf]
Exp. M$^+$ = 1068.4416
Calcd. M$^+$ = 1068.4447
2$_4$[OTf]
Exp. M$^+$ = 1312.5491
Calcd. M$^+$ = 1312.5527
1$_1$2$_1$[Br]
Exp. M$^+$ = 749.3069
Calcd. M$^+$ = 749.3086
1$_1$3$_1$[Br]
Exp. M$^+$ = 825.3375
Calcd. M$^+$ = 825.3399
2$_1$3$_1$[Br]
Exp. M$^+$ = 901.3642
Calcd. M$^+$ = 901.3712
1$_1$2$_1$[OTf]
Exp. M$^+$ = 749.3073
Calcd. M$^+$ = 749.3086
1$_1$3$_1$[OTf]
Exp. M$^+$ = 825.3415
Calcd. M$^+$ = 825.3399
2$_1$3$_1$[OTf]
Exp. M$^+$ = 901.3656
Calcd. M$^+$ = 901.3712
2$_1$Me[OTf]
Exp. M$^+$ = 520.2140
Calcd. M$^+$ = 520.2194
2$_3$Me[OTf]
Exp. M$^+$ = 1006.4277
Calcd. M$^+$ = 1006.4290
3$_3$Me[OTf]
Exp. M$^+$ = 1234.5177
Calcd. M$^+$ = 1234.5229
m/z

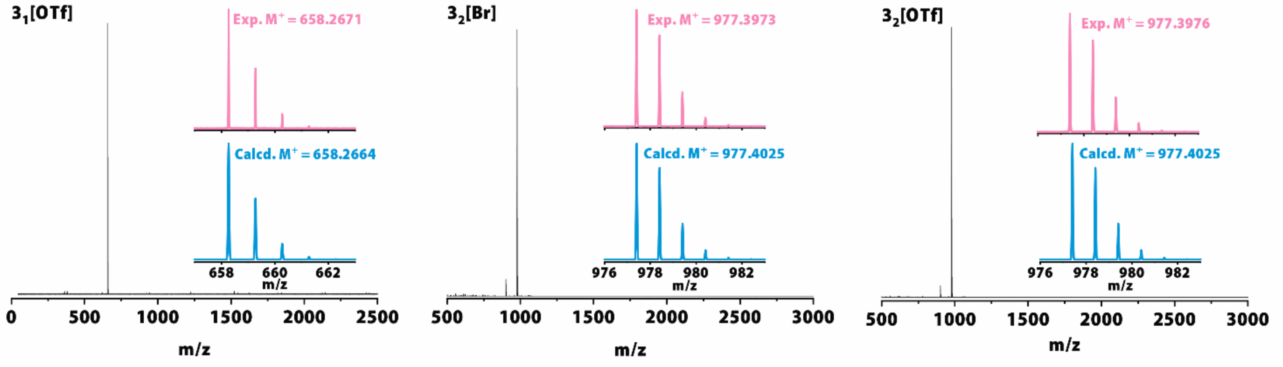

Figure S2. ESI$^+$ MS of salts ***Y$_n$Z$_n$***[X] and ***Y$_n$*Me**[X], calculated isotopic distributions are shown in blue.

Table S1. Photophysical data of $\boldsymbol{Y_nZ_n}$[X] and $\boldsymbol{Y_n}$**Me**[X] salts in dichloromethane and acetonitrile at 298 K.

Cation	Anion A⁻	Solvent	λ_{abs}, nm<br>(ε, 10^{-3}·M^{-1}·cm^{-1})	λ_{em}, nm	Stokes shift, cm^{-1}	Φ_{em}	τ, ns	k_r,[a] 10^7 s^{-1}	k_{nr},[b] 10^7 s^{-1}
**2_1**	Br⁻	DCM<br>MeCN	387 (27.2)<br>372 (26.8)	528<br>555	6900<br>8864	0.88<br>0.51	4.4<br>4.1	20.0<br>12.4	2.7<br>4.9
**2_2**	Br⁻	DCM<br>MeCN	390 (43.6)<br>375 (40.4)	528<br>550	6700<br>8485	0.73<br>0.39	4.4<br>3.2	16.5<br>12.1	6.1<br>19.1
	OTf⁻	DCM<br>MeCN	392 (44.0)<br>374 (40.8)	527<br>551	6635<br>8589	0.76<br>0.34	4.4<br>3.3	17.3<br>10.3	5.4<br>20.0
**2_3**	Br⁻	DCM<br>MeCN	392 (62.8)<br>378 (58.4)	523<br>549	6390<br>8241	0.76<br>0.34	4.2<br>2.9	18.1<br>11.7	5.7<br>22.7
	OTf⁻	DCM<br>MeCN	393 (63.3)<br>377 (59.0)	525<br>549	6408<br>8310	0.81<br>0.29	4.2<br>2.9	19.3<br>10.0	4.5<br>24.5
**2_4**	Br⁻	DCM<br>MeCN	393 (84.2)<br>378 (78.7)	522<br>547	6288<br>7174	0.79<br>0.30	4.1<br>2.7	19.2<br>11.1	5.1<br>25.9
	OTf⁻	DCM<br>MeCN	394 (85.2)<br>378 (79.0)	523<br>549	6260<br>7241	0.77<br>0.25	4.1<br>2.7	18.8<br>9.2	5.6<br>27.8
**3_1**	OTf⁻	DCM<br>MeCN	298 (36.6), 378 (34.4)<br>286 (40.4), 363 (38.8)	573<br>617	9003<br>11341	0.59<br>0.23	4.4<br>2.6	13.4<br>8.8	9.3<br>29.6
**3_2**	Br⁻	DCM<br>MeCN	296, 378<br>288, 364	575<br>620	9064<br>11343	0.26<br>0.06	4.0<br>0.9	6.5<br>6.7	18.5<br>104.4
	OTf⁻	DCM<br>MeCN	296 (78.9), 381 (60.2)<br>288 (64.7), 365 (63.0)	575<br>617	8855<br>10390	0.30<br>0.06	4.0<br>0.9	7.5<br>6.7	17.5<br>104.4
**1_12_1**	Br⁻	DCM<br>MeCN	380<br>363	517<br>543	6973<br>9132	0.93<br>0.67	4.0<br>4.2	23.4<br>16.1	1.8<br>7.9
	OTf⁻	DCM<br>MeCN	375 (39.7)<br>361 (35.9)	520<br>542	7436<br>9251	0.96<br>0.67	4.0<br>4.1	24.2<br>16.2	1.0<br>8.0
**1_13_1**	Br⁻	DCM<br>MeCN	293, 359<br>287, 354	564<br>610	10125<br>11855	0.77<br>0.32	3.7<br>2.8	20.8<br>11.4	6.2<br>24.0
	OTf⁻	DCM<br>MeCN	294 (54.5), 362 (51.5)<br>288 (44.4), 353 (49.2)	566<br>609	9957<br>11908	0.79<br>0.33	3.7<br>2.8	21.4<br>11.7	5.7<br>23.9
**2_13_1**	Br⁻	DCM<br>MeCN	300, 383<br>286, 366	572<br>605	8627<br>10794	0.66<br>0.16	3.9<br>2.0	16.9<br>8.0	8.7<br>42.0
	OTf⁻	DCM<br>MeCN	295 (48.5), 385 (54.7)<br>287 (45.2), 367 (57.6)	570<br>603	8431<br>10664	0.67<br>0.15	3.9<br>2.0	17.2<br>7.5	8.4<br>42.5
**2_1Me**	OTf⁻	DCM<br>MeCN	386 (30.6)<br>367 (29.6)	525<br>541	6859<br>8763	0.87<br>0.58	4.4<br>4.3	19.8<br>13.4	3.0<br>9.8
**2_3Me**	OTf⁻	DCM<br>MeCN	389 (68.2)<br>372 (65.1)	519<br>540	6439<br>8363	0.81<br>0.41	4.3<br>3.2	18.8<br>12.8	4.4<br>18.4
**3_3Me**	OTf⁻	DCM<br>MeCN	296 (105.1), 379 (92.7)<br>288 (82.4), 362 (89.9)	571<br>600	8872<br>10598	0.59<br>0.05	4.3<br>1.0	13.7<br>5.0	9.5<br>95.0

[a] k_r values were estimated by Φ_{em}/τ; [b] k_{nr} values were estimated by $(1 - \Phi)/\tau$.

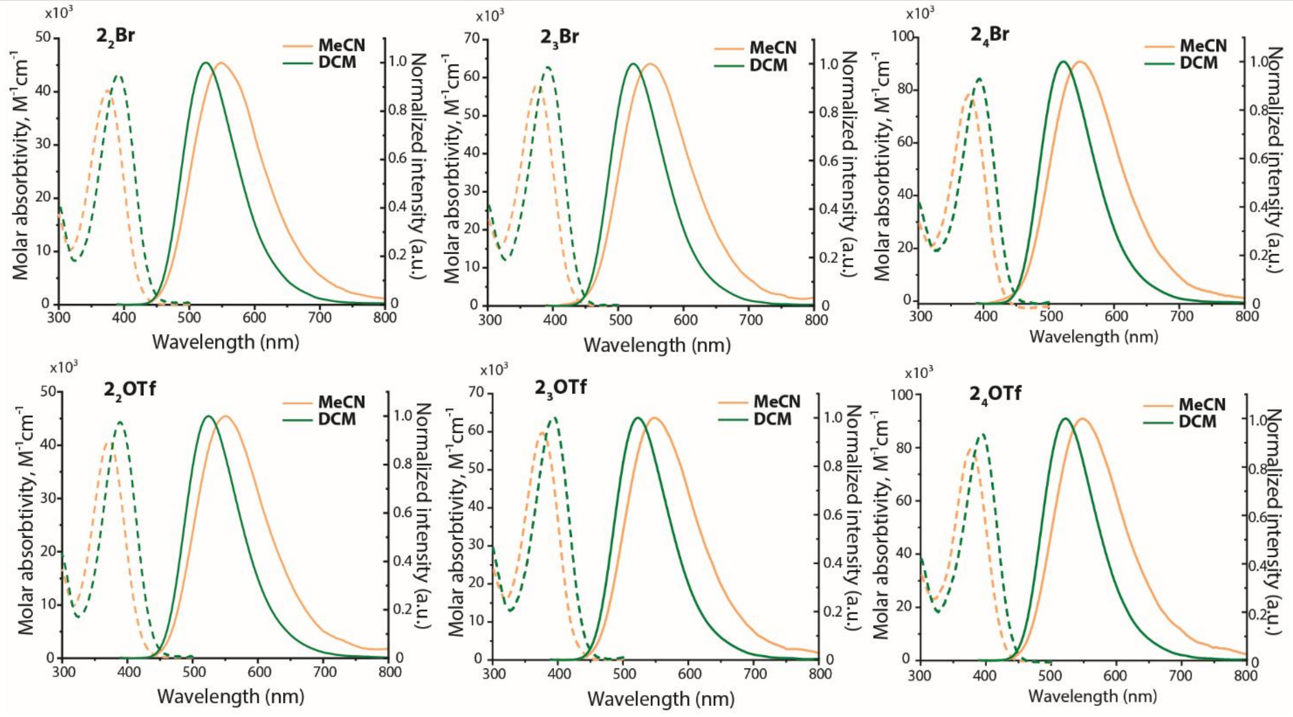

Figure S3. Absorption (dashed lines) and normalized emission (solid lines) spectra of salts $\mathbf{2}_n$[X] (n = 2–4; A^- = Br^-, OTf^-) in dichloromethane (DCM) and acetonitrile (MeCN) at 298 K.

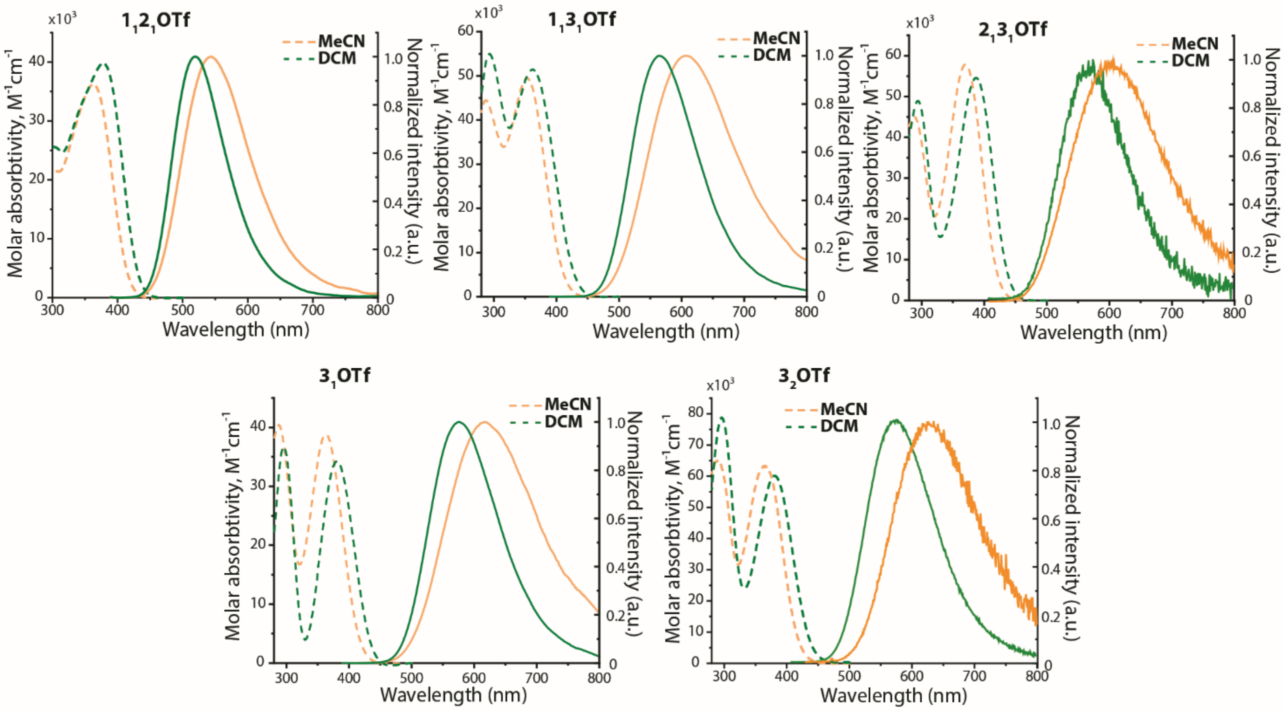

Figure S4. Absorption (dashed lines) and normalized emission (solid lines) spectra of salts $\mathbf{1}_1\mathbf{2}_1$[OTf], $\mathbf{1}_1\mathbf{3}_1$[OTf], $\mathbf{2}_1\mathbf{3}_1$[OTf] and $\mathbf{3}_2$[OTf] in dichloromethane and acetonitrile at 298 K.

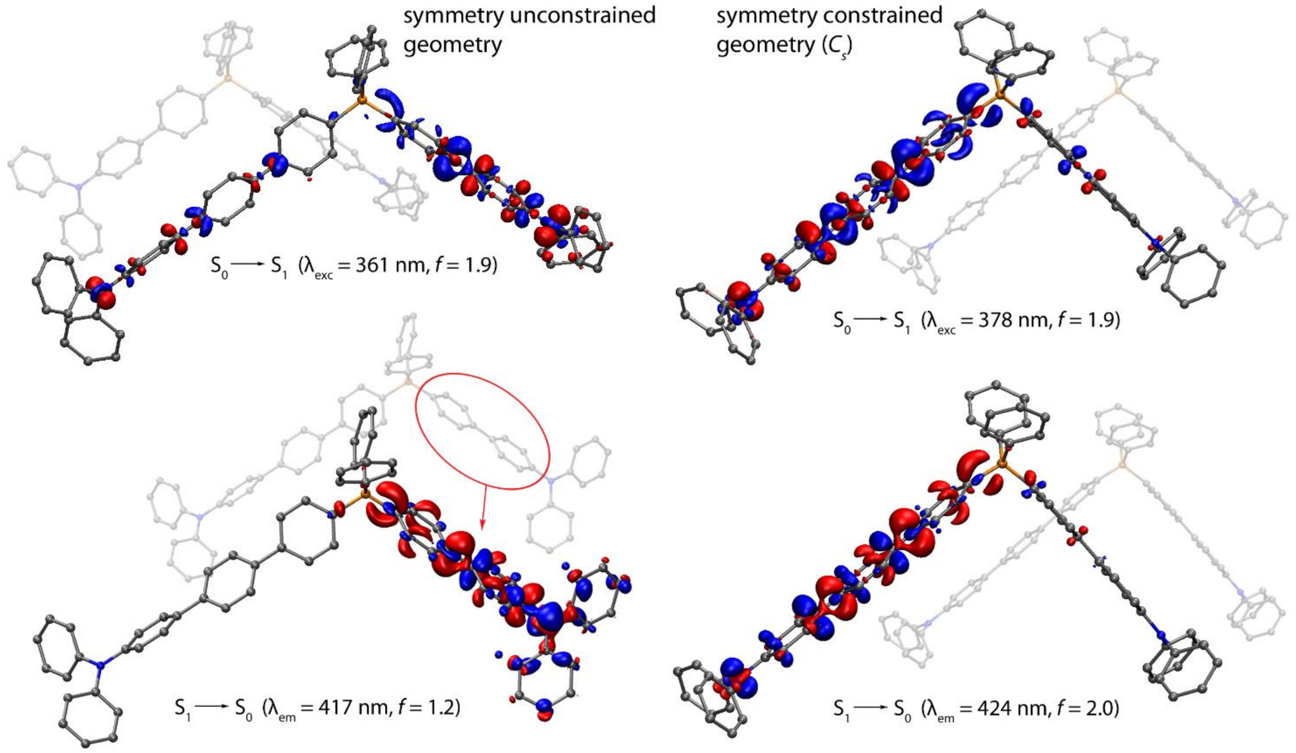

Figure S5. Lowest energy excitation $S_0 \rightarrow S_1$ and emission $S_1 \rightarrow S_0$ electron density difference plots for cation **2₁3₁**$^+$ (isovalue 0.002 a.u., DFT-LRC-ωPBEh, optimized S_0 and S_1 geometries shown in semi-transparent mode), calculated for symmetry unconstrained (left) and symmetry constrained (right, C_s, an averaged conformation) geometries. During the electronic transition, the electron density increases in the blue areas and decreases in the red areas. Hydrogen atoms and counterions are omitted for clarity.

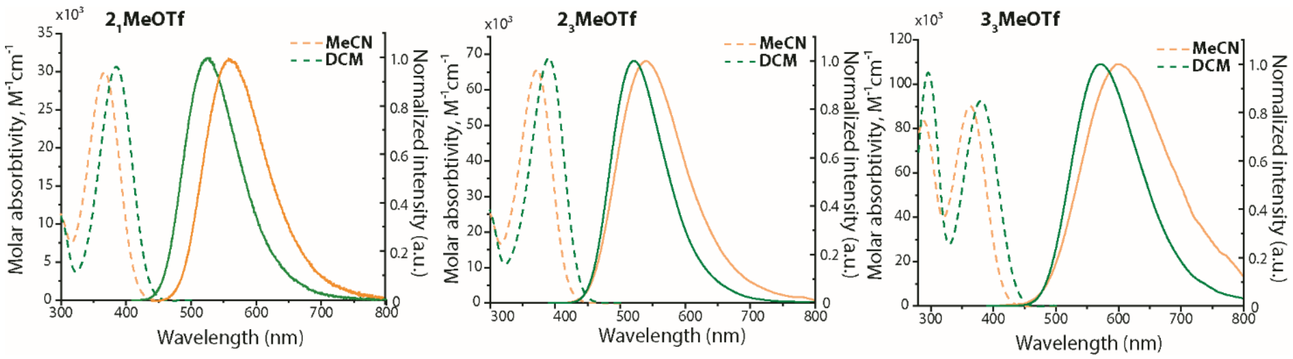

Figure S6. Absorption (dashed lines) and normalized emission (solid lines) spectra of salts $\mathbf{2_1Me}$[OTf], $\mathbf{2_3Me}$[OTf] and $\mathbf{3_3Me}$[OTf] in dichloromethane and acetonitrile at 298 K.

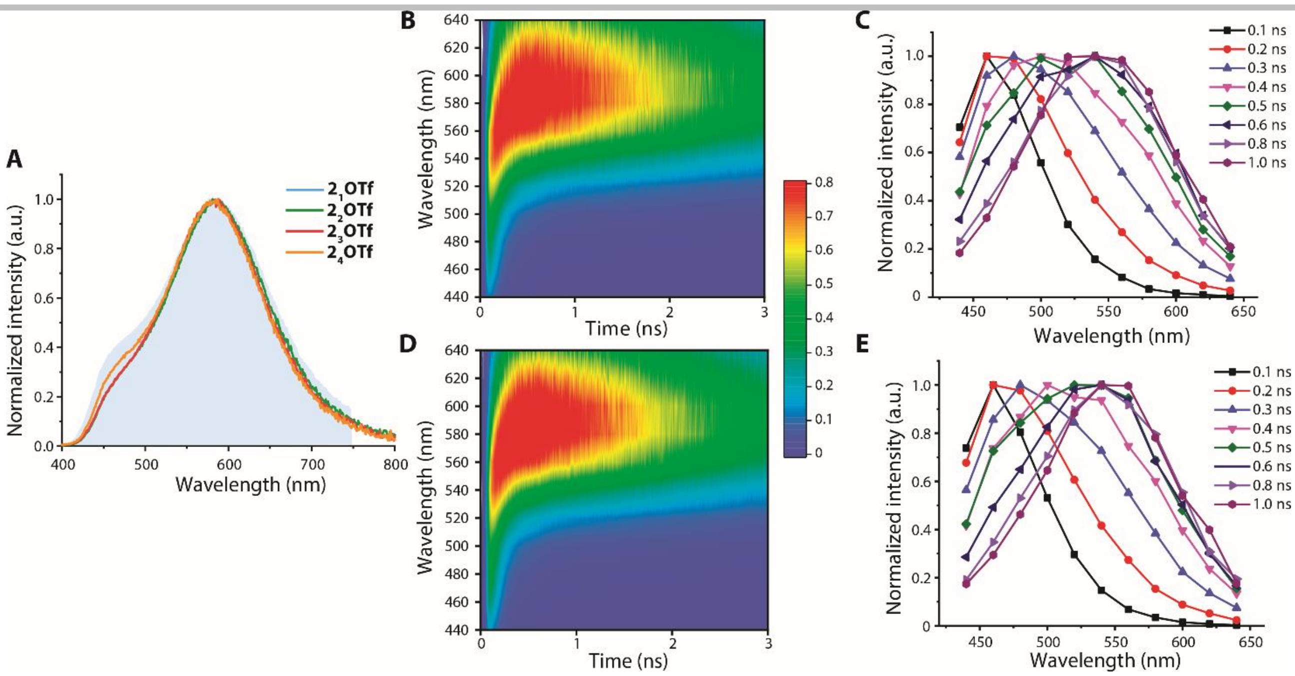

Figure S7. Normalized emission spectra of $\mathbf{2}_n$[OTf] n = 1–4 (A), time-resolved emission spectra of $\mathbf{2}_3$[OTf] (B, C) and $\mathbf{2}_4$[OTf] (D, E) in toluene at 298 K.

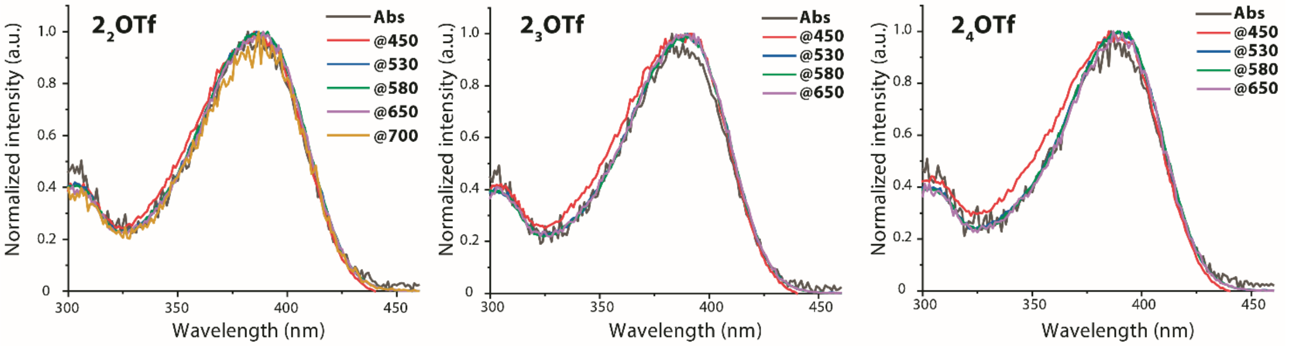

Figure S8. Normalized absorption and excitation spectra of $\mathbf{2}_n$[OTf] n = 2–4 in toluene at 298 K.

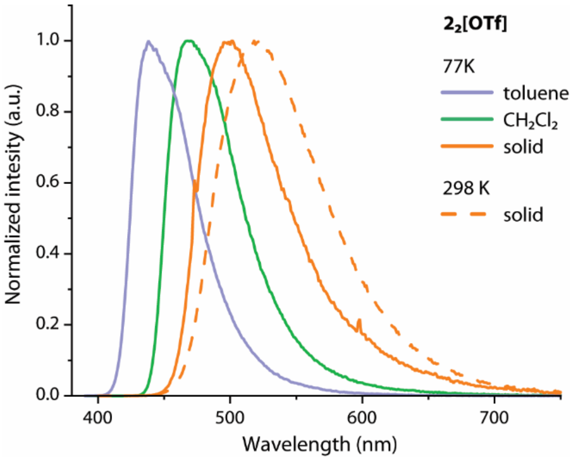

Figure S9. Normalized emission spectra of $\mathbf{2}_2$[OTf] in frozen glass of toluene and dichloromethane at 77 K, and in solid state at 298 K and 77 K.

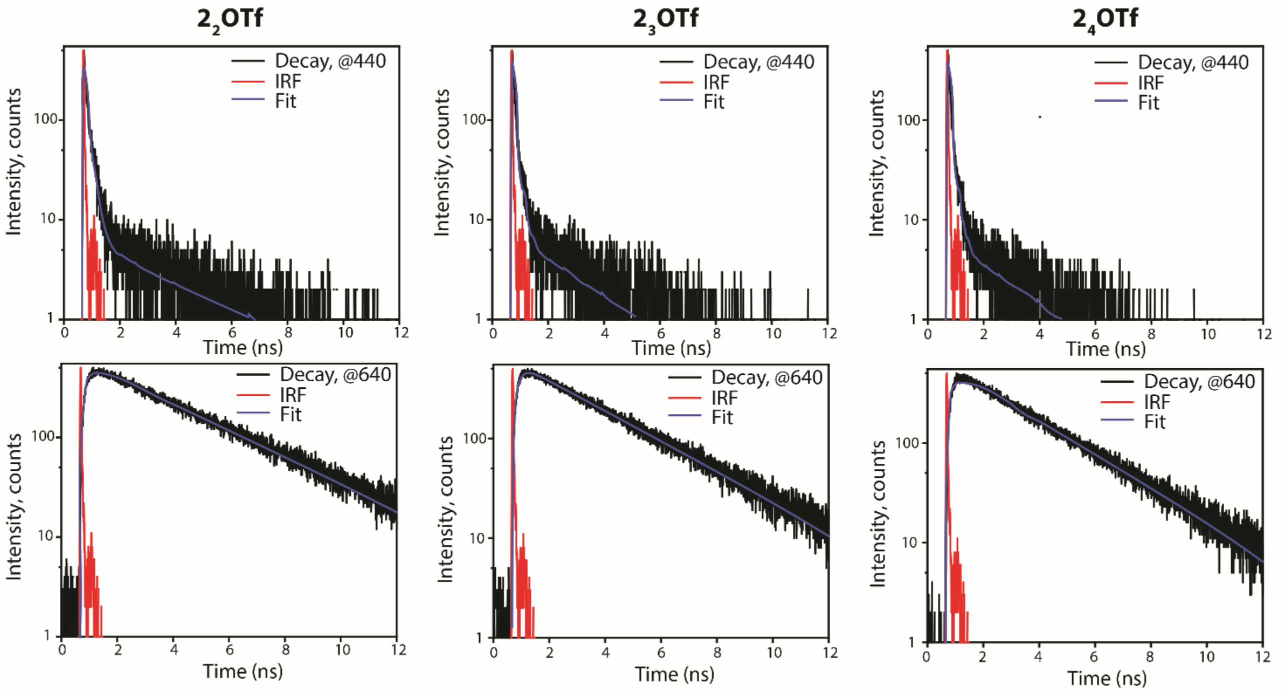

Figure S10. Emission decay profiles of **2*$_n$***[OTf] *n* = 2–4 monitored at 440 nm (F_1 band, top) and 640 nm (F_2 band, bottom) in toluene at 298 K.

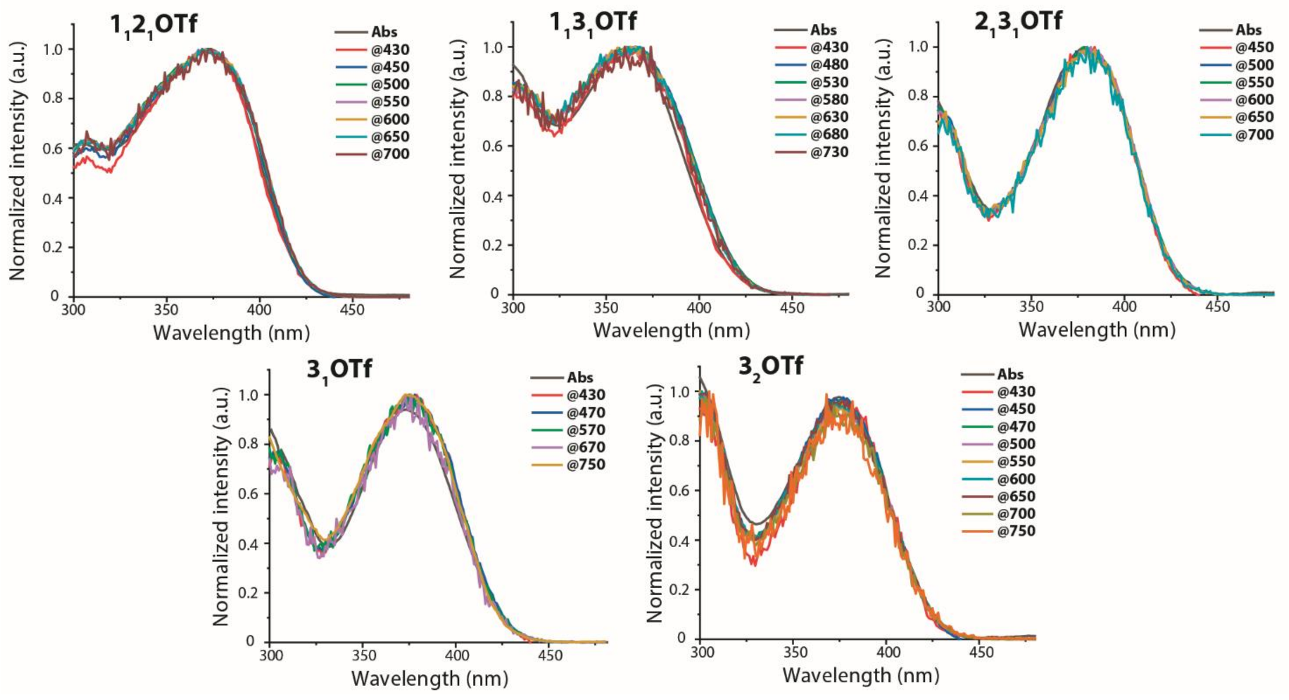

Figure S11. Normalized absorption and excitation spectra of **1**$_1$**2**$_1$[OTf], **1**$_1$**3**$_1$[OTf], **2**$_1$**3**$_1$[OTf] and **3**$_2$[OTf] in toluene at 298 K.

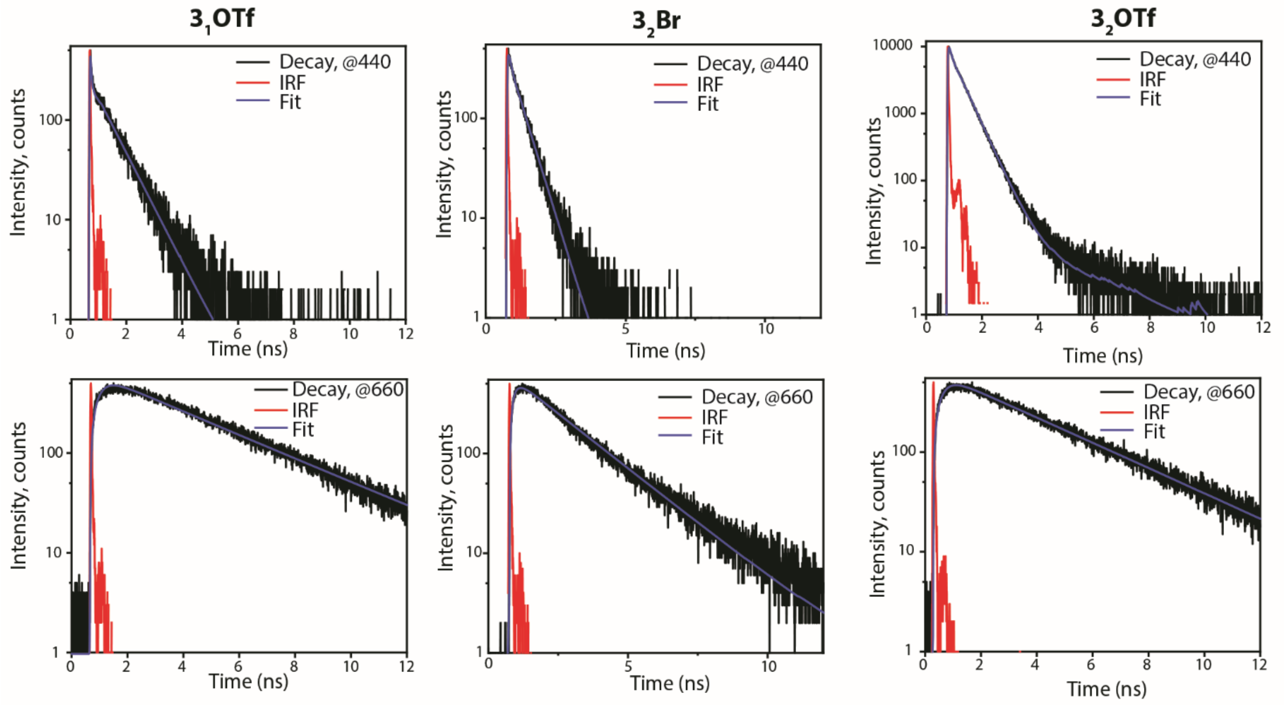

Figure S12. Emission decay profiles of **3**$_1$[OTf], **3**$_2$[Br] and **3**$_2$[OTf] monitored at 440 nm (F_1 band, top) and 660 nm (F_2 band, bottom) in toluene at 298 K.

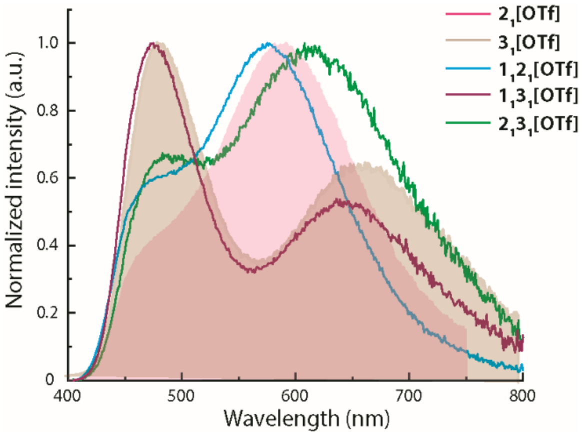

Figure S13. Normalized emission spectra of **1**$_1$**2**$_1$[OTf], **1**$_1$**3**$_1$[OTf] and **2**$_1$**3**$_1$[OTf] in toluene at 298 K, (filled graphs correspond to **2**$_1$[OTf] and **3**$_1$[OTf] for comparison).

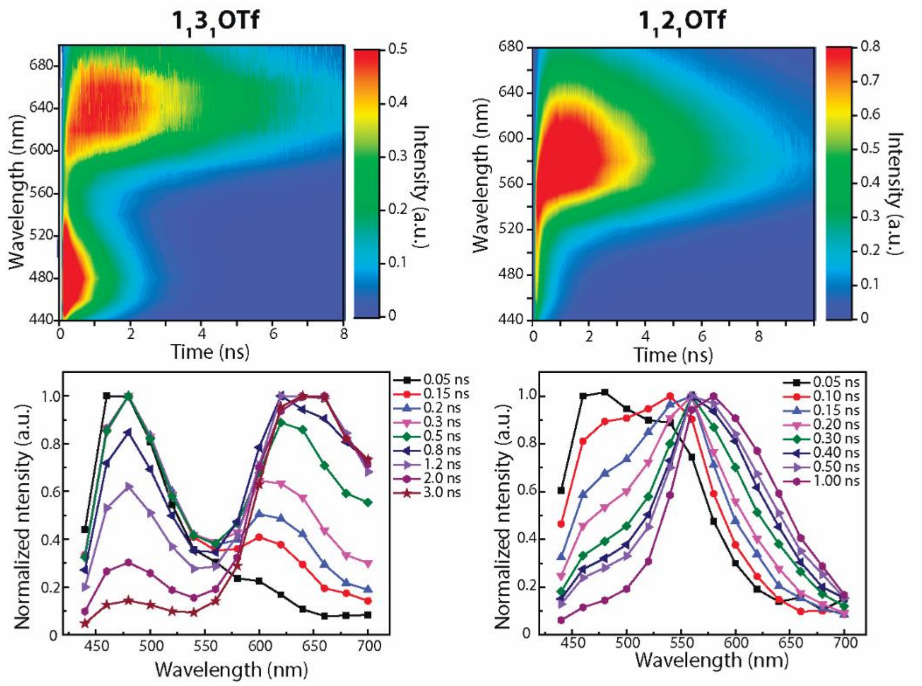

Figure S14. Time-resolved emission spectra of **1**$_1$**2**$_1$[OTf] and **1**$_1$**3**$_1$[OTf] in toluene, 298 K.

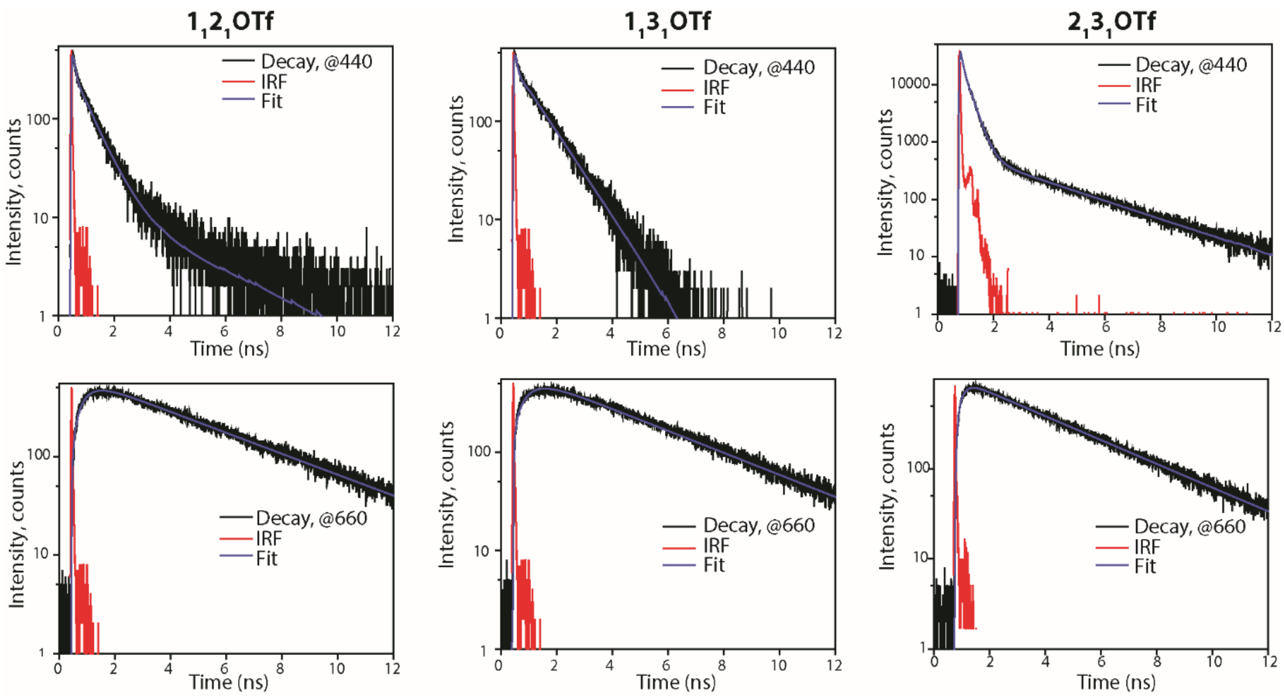

Figure S15. Emission decay profiles of **1**$_1$**2**$_1$[OTf], **1**$_1$**3**$_1$[OTf], **2**$_1$**3**$_1$[OTf] monitored at 440 nm (top) and 660 nm (bottom) in toluene at 298 K.

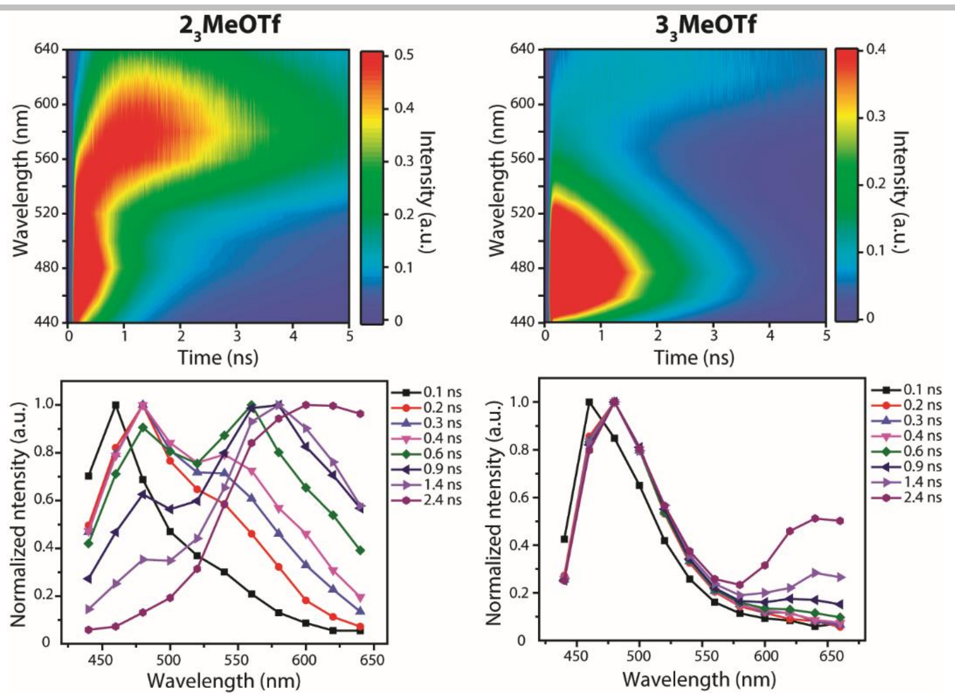

Figure S16. Time-resolved emission spectra of **2_3Me**[OTf] and **3_3Me**[OTf] in toluene, 298 K.

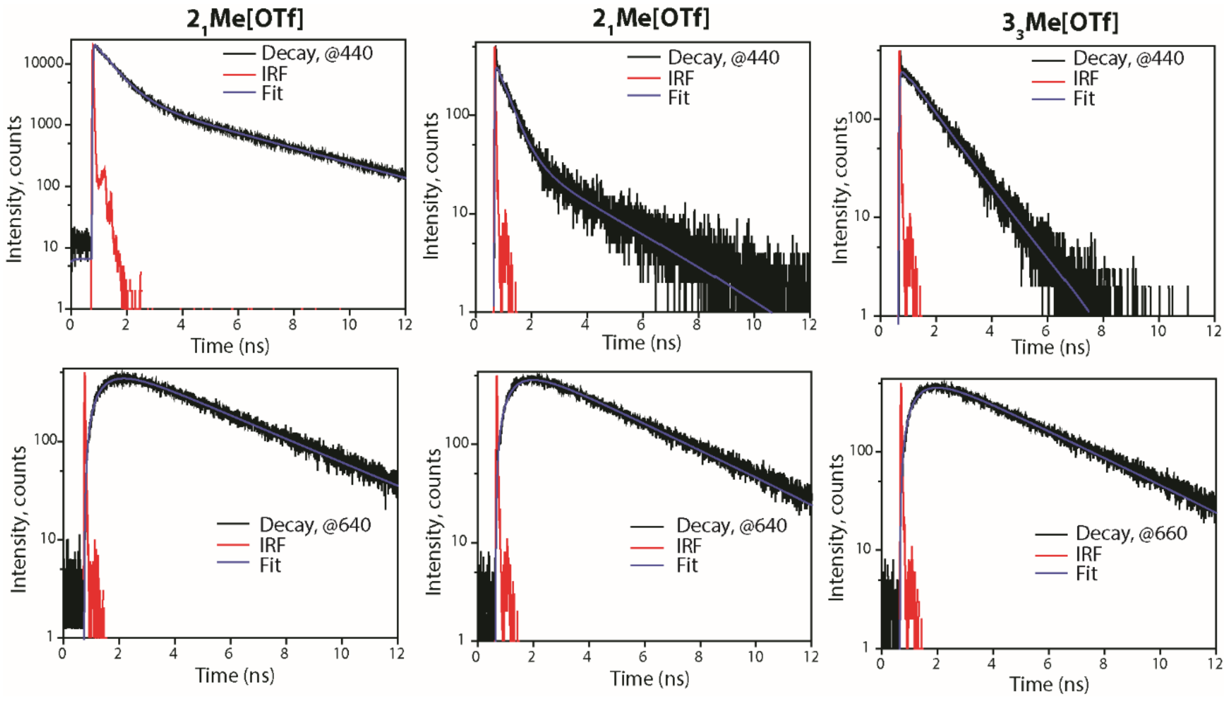

Figure S17. Emission decay profiles of **2_1Me**[OTf], **2_3Me**[OTf] and **3_3Me**[OTf] monitored at 440 nm (F_1 band, top) and 660 nm (F_2 band, bottom) in toluene at 298 K.

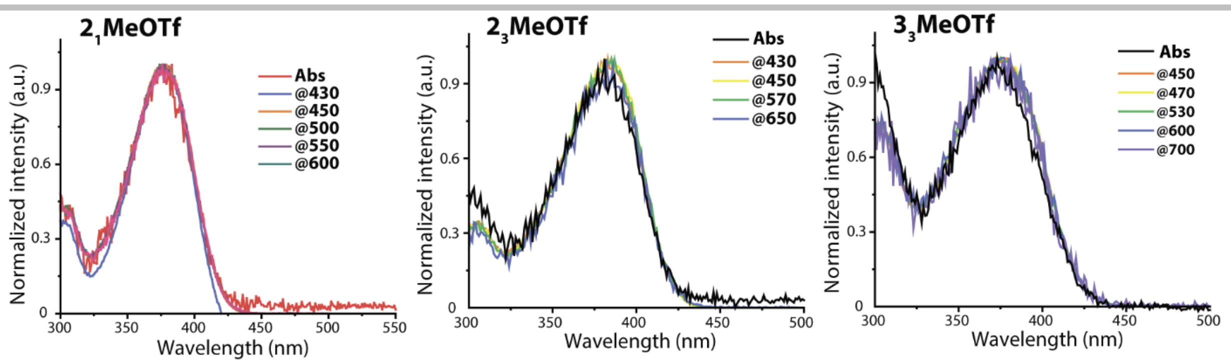

Figure S18. Normalized absorption and excitation spectra of **2_1Me**[OTf], **2_3Me**[OTf] and **3_3Me**[OTf] in toluene at 298 K.

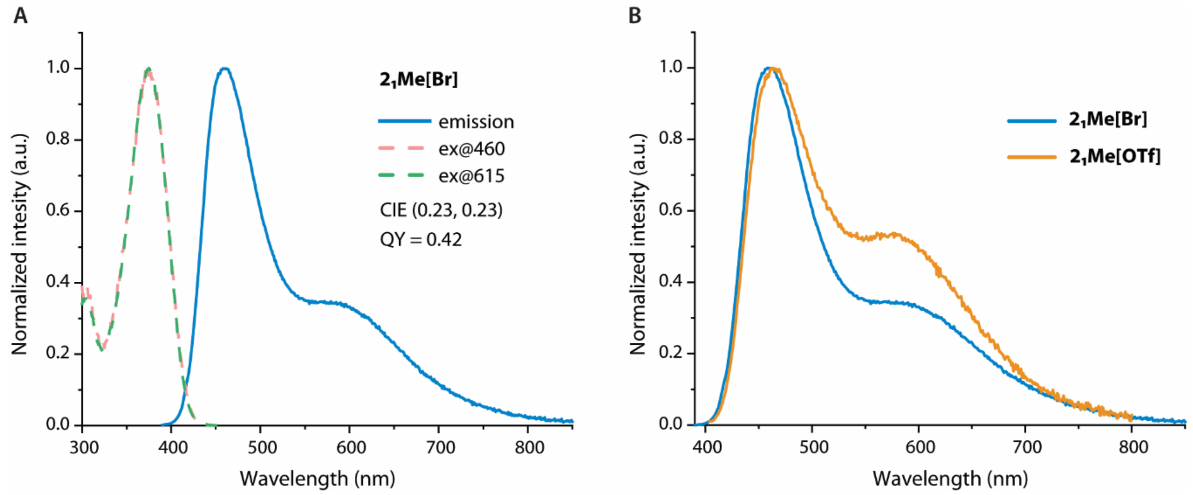

Figure S19. (A) Normalized excitation and emission spectra of **2_1Me**[Br] in toluene at 298 K; (B) normalized at HiE band emission spectra of **2_1Me**[Br] and **2_1Me**[OTf] in toluene at 298 K.

NMR spectra

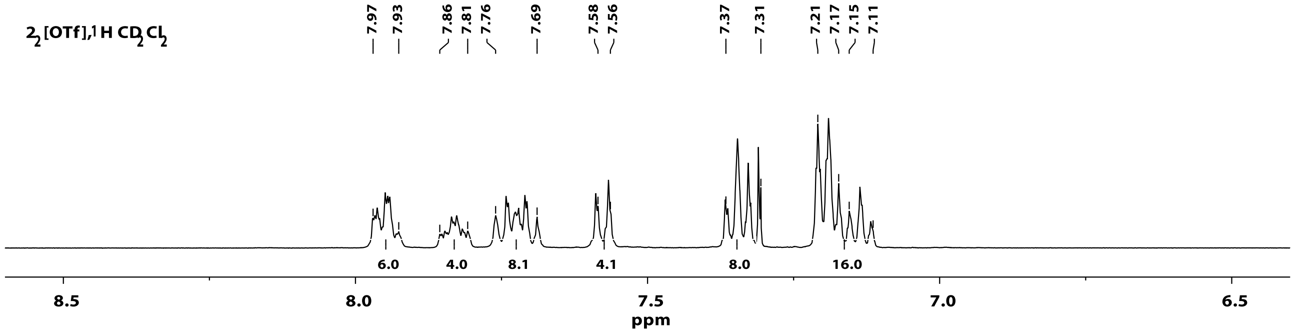
2_2[OTf],1H CD$_2$Cl$_2$

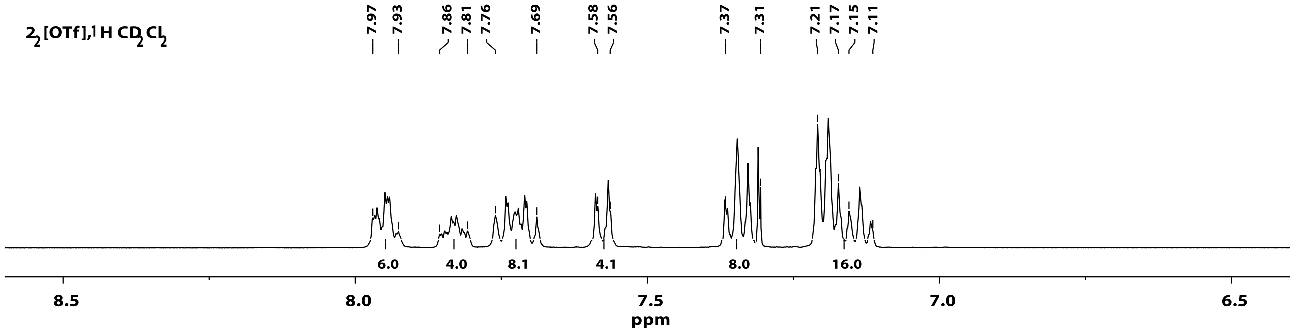
2_2[OTf],1H CD$_2$Cl$_2$

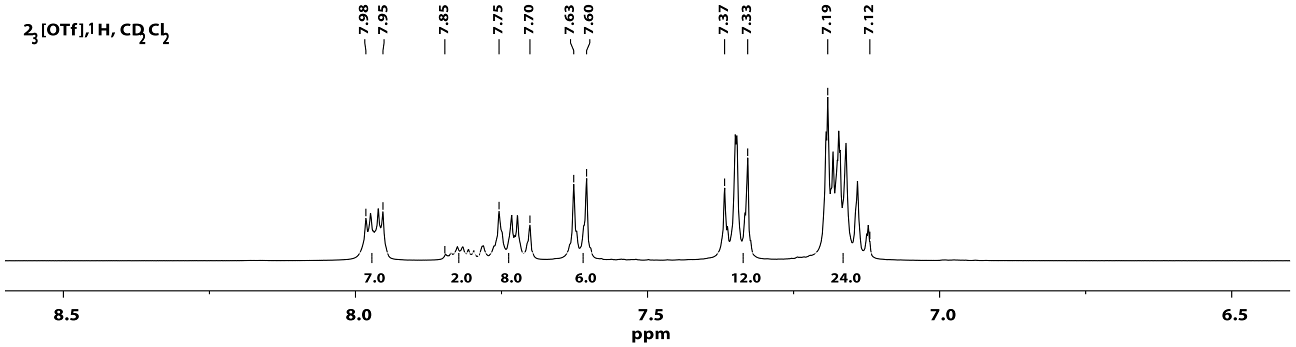
2_3[OTf],1H, CD$_2$Cl$_2$

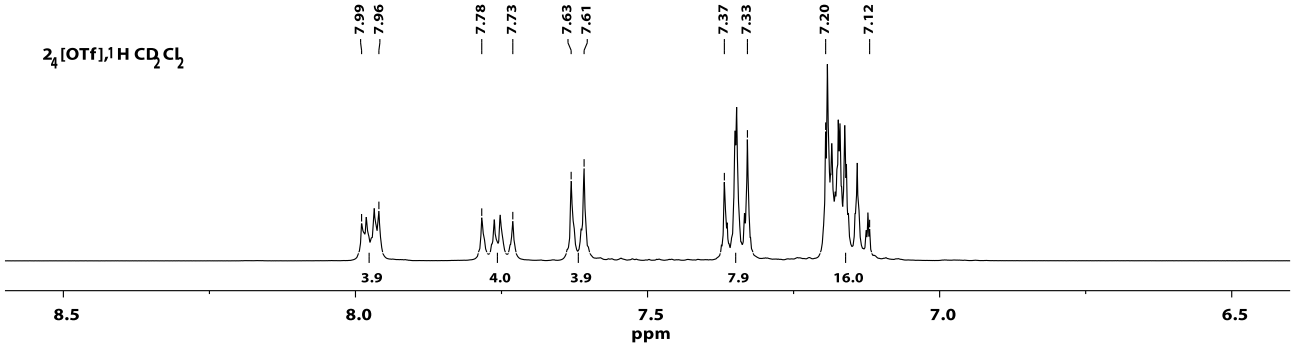
2_4[OTf],1H CD$_2$Cl$_2$

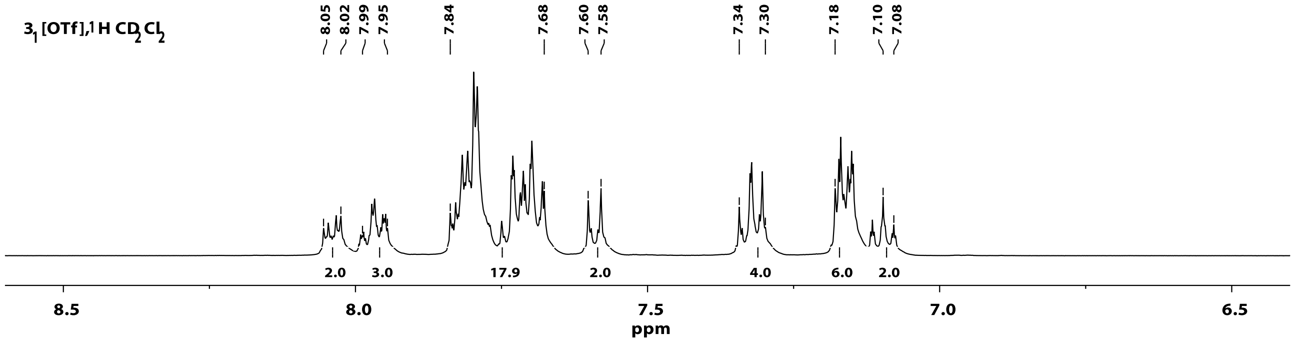
3_1[OTf],1H CD$_2$Cl$_2$

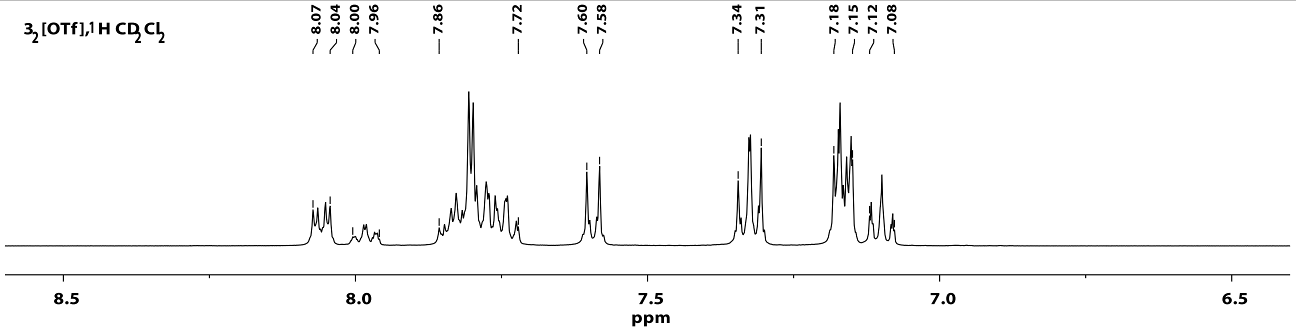
3$_2$[OTf]$_2$ ^{1}H CD$_2$Cl$_2$
8.07
8.04
8.00
7.96
7.86
7.72
7.60
7.58
7.34
7.31
7.18
7.15
7.12
7.08
8.5
8.0
7.5
7.0
6.5
ppm

$1_1 2_1$[OTf], 1H CD_2Cl_2

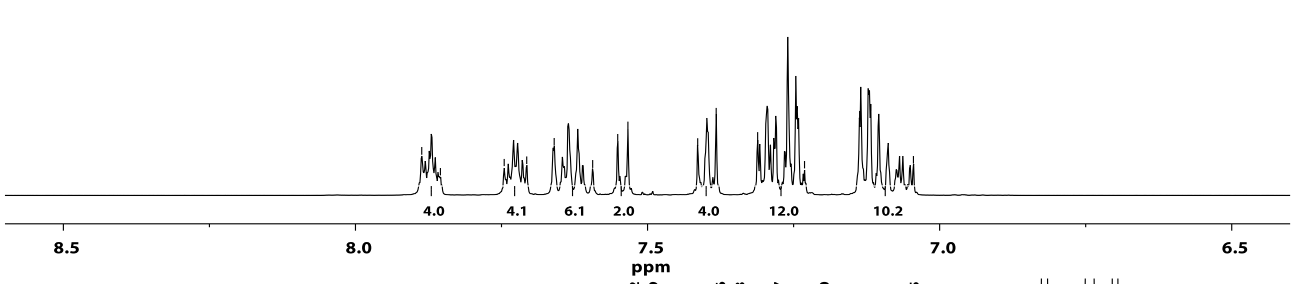

$1_1 2_1$[OTf], 1H, Toluene-D8

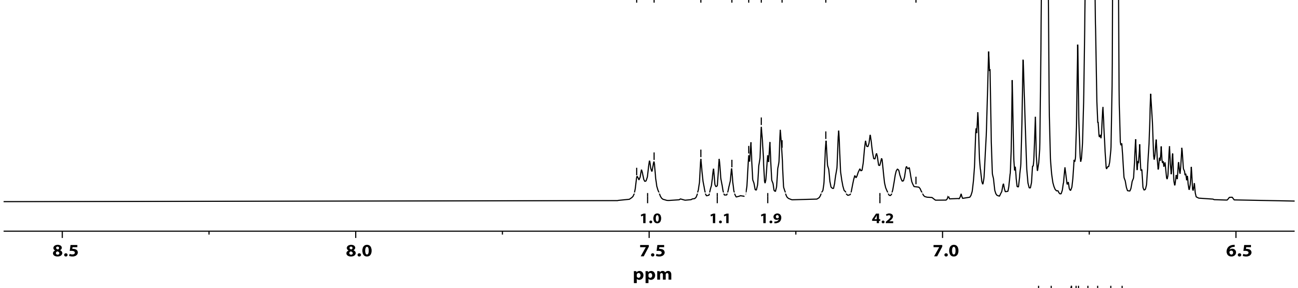

$1_1 2_1$[Br], 1H, Toluene-D8

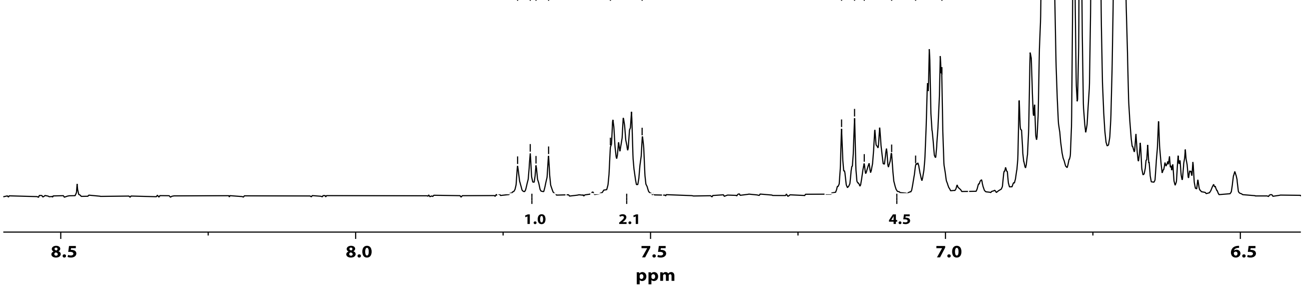

$1_1 3_1$[OTf], 1H CD_2Cl_2

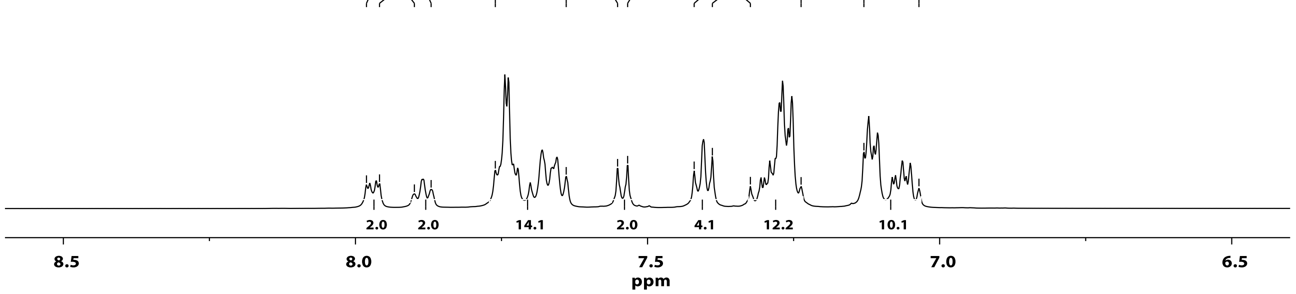

$2_1 3_1$[OTf], 1H CD_2Cl_2

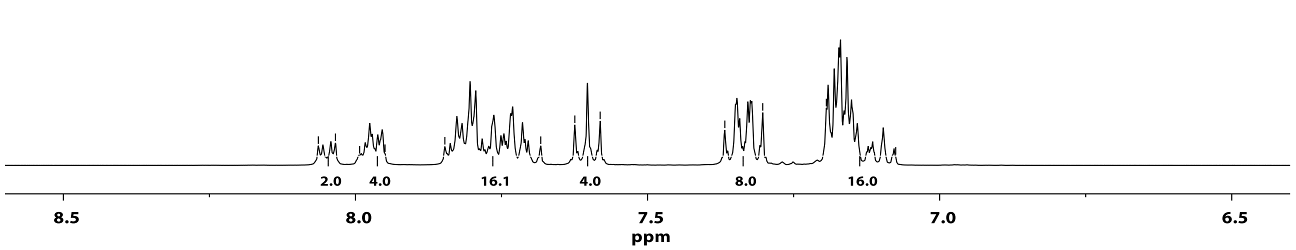

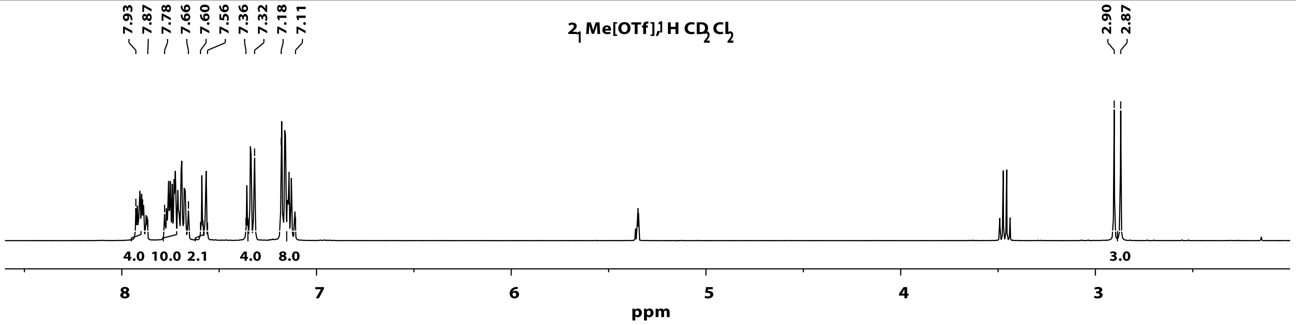
2₁ Me[OTf], 1H CD2Cl2
7.93 7.87 7.78 7.66 7.60 7.56 7.36 7.32 7.18 7.11
2.90 2.87
4.0 10.0 2.1 4.0 8.0 3.0
8 7 6 5 4 3
ppm

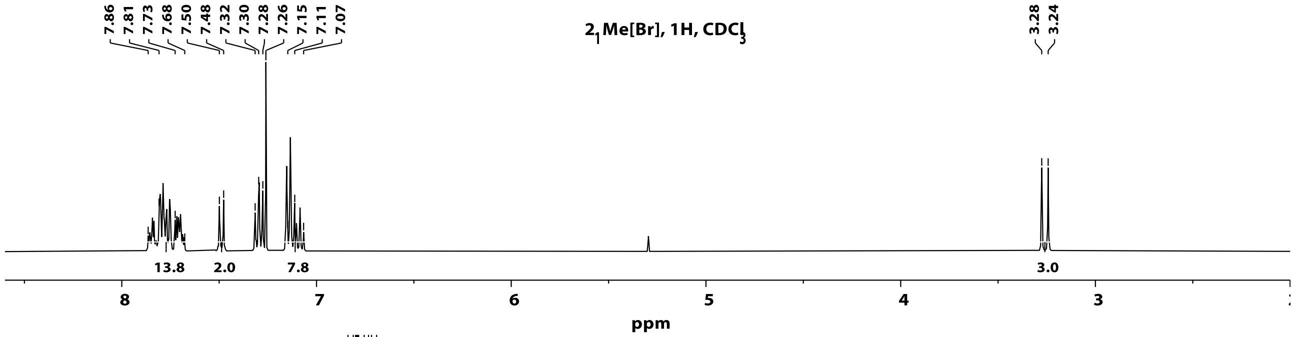
2₁ Me[Br], 1H, CDCl3
7.86 7.81 7.73 7.68 7.50 7.48 7.32 7.30 7.28 7.26 7.15 7.11 7.07
3.28 3.24
13.8 2.0 7.8 3.0
8 7 6 5 4 3
ppm

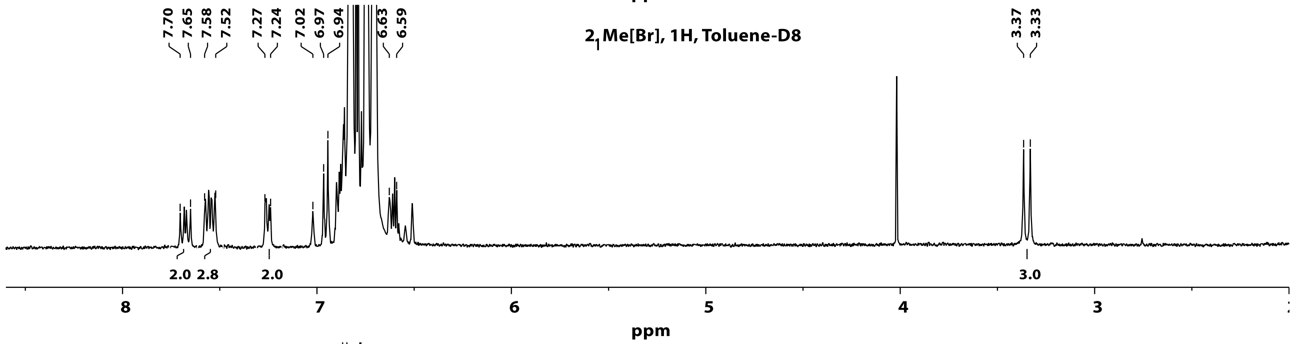
2₁ Me[Br], 1H, Toluene-D8
7.70 7.65 7.58 7.52 7.27 7.24 7.02 6.97 6.94 6.63 6.59
3.37 3.33
2.0 2.8 2.0 3.0
8 7 6 5 4 3
ppm

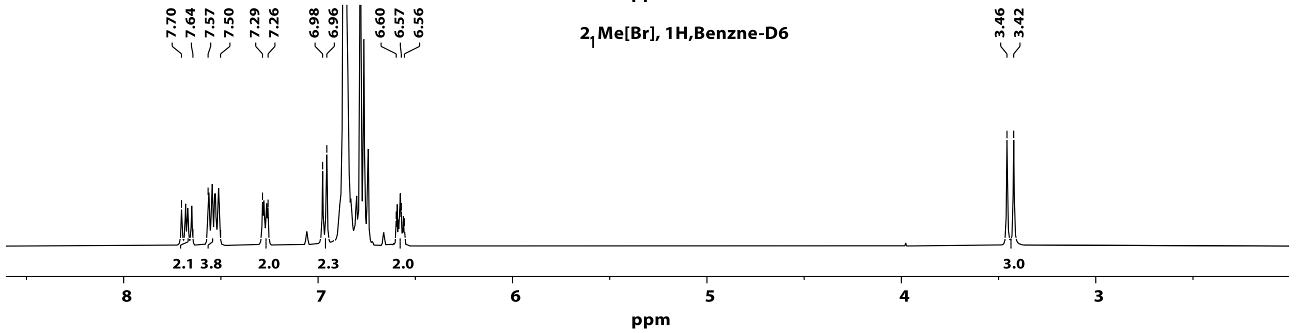
2₁ Me[Br], 1H,Benzne-D6
7.70 7.64 7.57 7.50 7.29 7.26 6.98 6.96 6.60 6.57 6.56
3.46 3.42
2.1 3.8 2.0 2.3 2.0 3.0
8 7 6 5 4 3
ppm

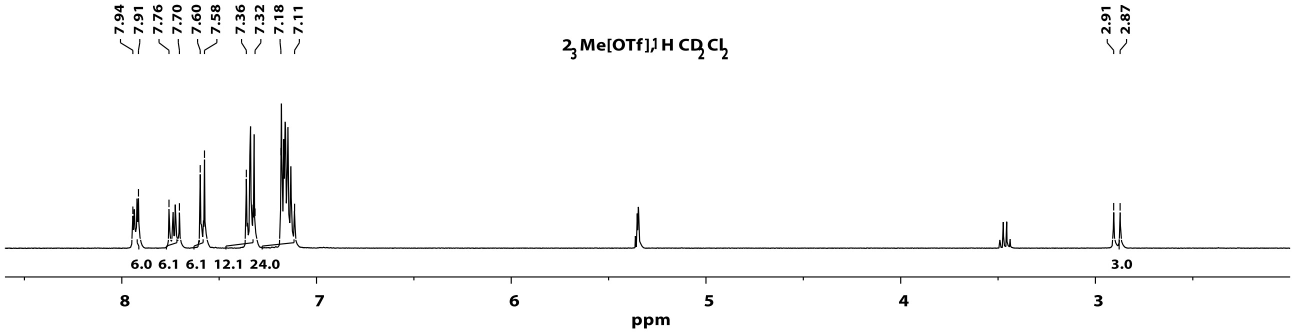
2₃ Me[OTf], 1H CD2Cl2
7.94 7.91 7.76 7.70 7.60 7.58 7.36 7.32 7.18 7.11
2.91 2.87
6.0 6.1 6.1 12.1 24.0 3.0
8 7 6 5 4 3
ppm

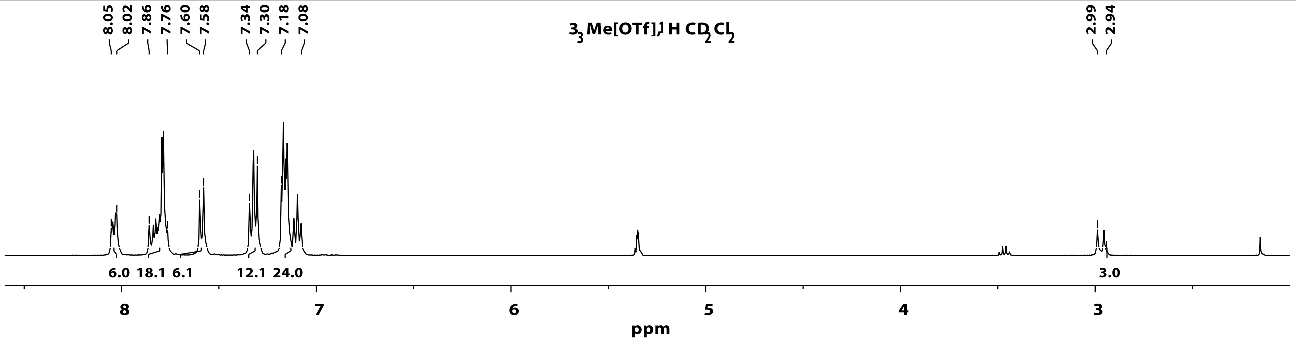
8.05
8.02
7.86
7.76
7.60
7.58
7.34
7.30
7.18
7.08
3, Me[OTf], 1H CD2Cl2
2.99
2.94
6.0 18.1 6.1 12.1 24.0
3.0
8
7
6
5
4
3
ppm

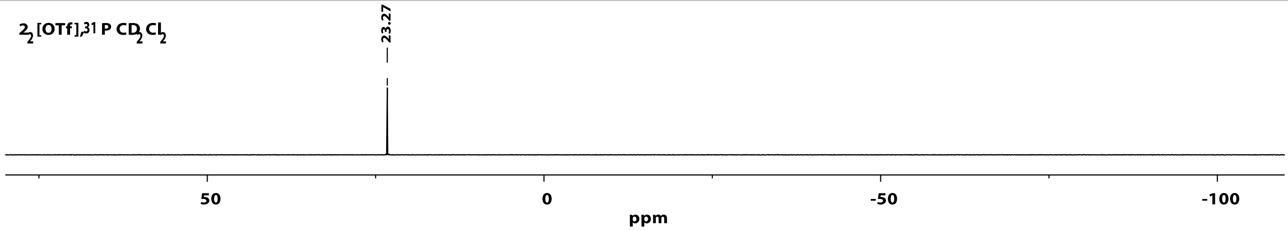
2_2[OTf],31 P CD_2Cl_2
23.27
50
0
-50
-100
ppm

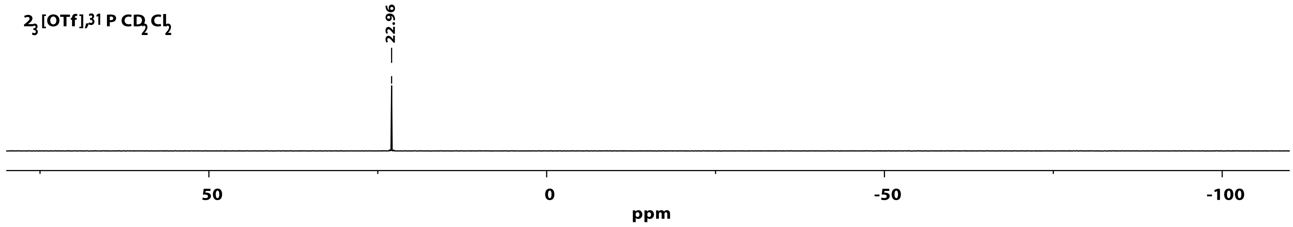
2_3[OTf],31 P CD_2Cl_2
22.96
50
0
-50
-100
ppm

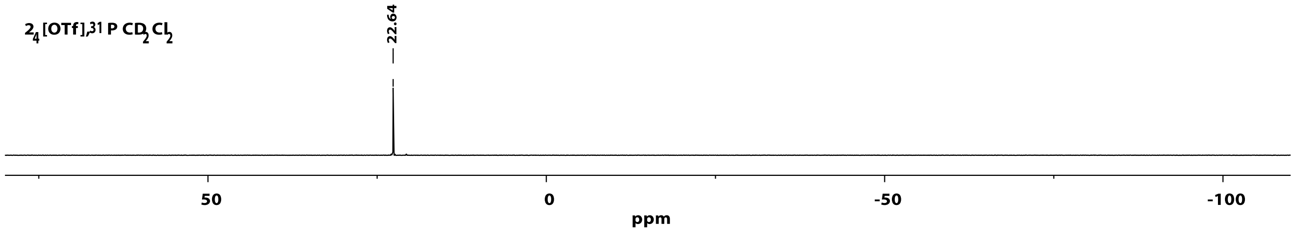
2_4[OTf],31 P CD_2Cl_2
22.64
50
0
-50
-100
ppm

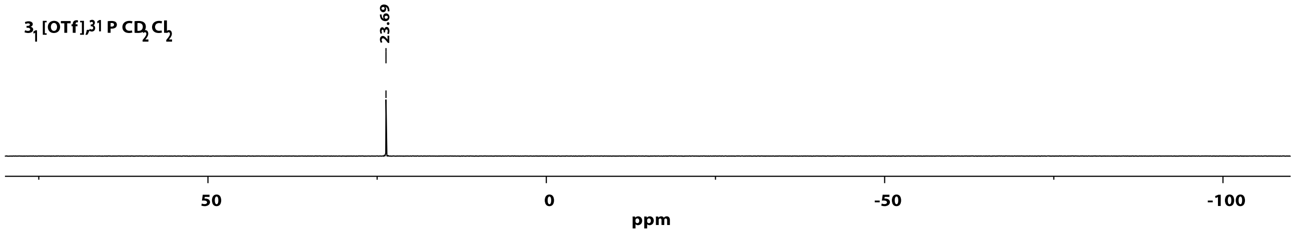
3_1[OTf],31 P CD_2Cl_2
23.69
50
0
-50
-100
ppm

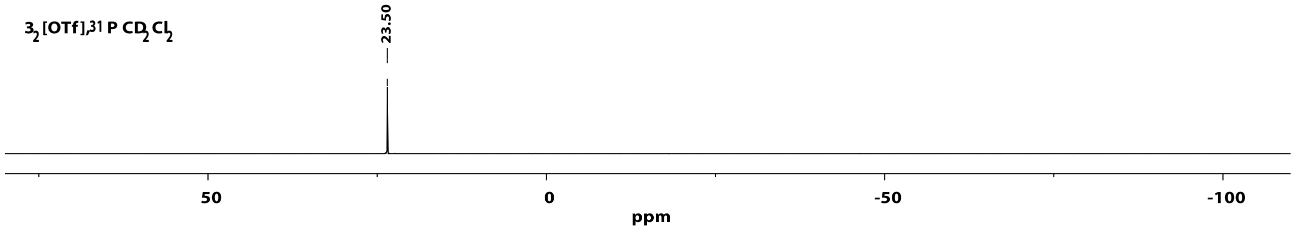
3_2[OTf],31 P CD_2Cl_2
23.50
50
0
-50
-100
ppm

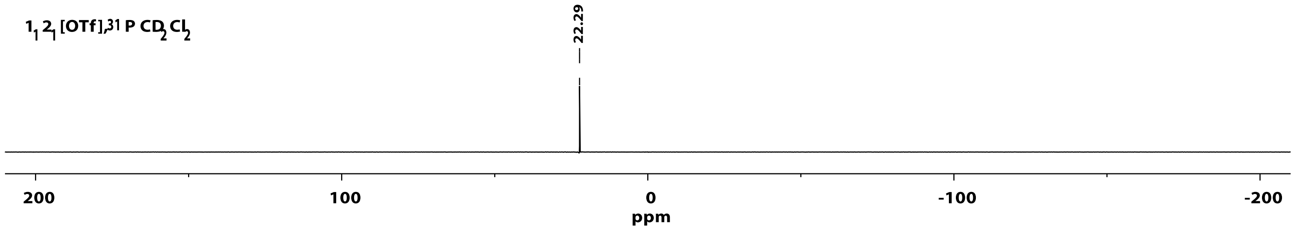
$1_1 2_1$[OTf],31 P CD_2Cl_2
22.29
200
100
0
-100
-200
ppm

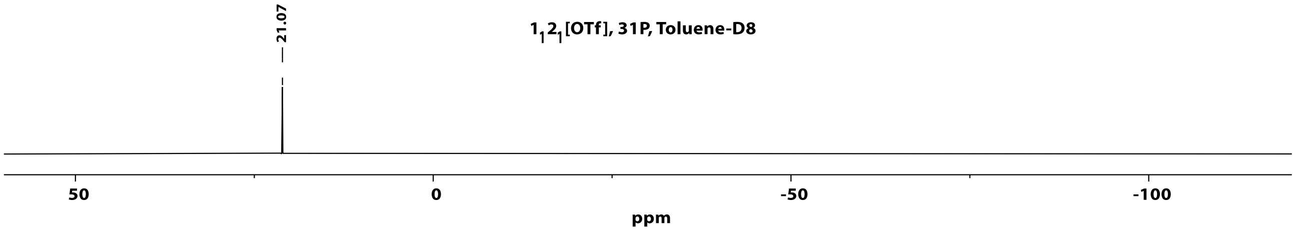
21.07
$1_1 2_1$[OTf], 31P, Toluene-D8
50
0
-50
-100
ppm

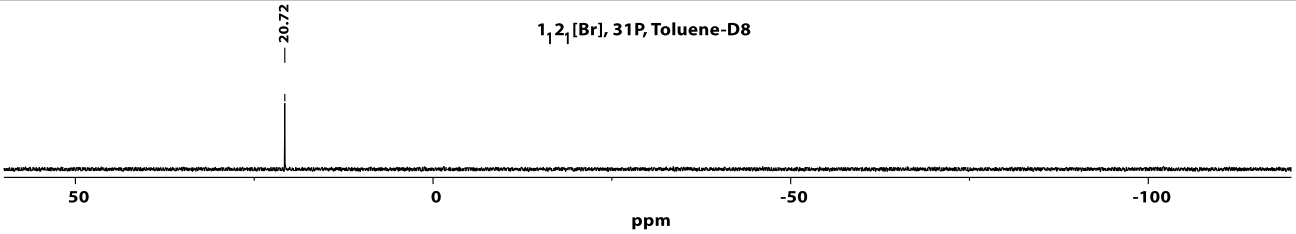
20.72
$1_1 2_1$[Br], 31P, Toluene-D8
50
0
-50
-100
ppm

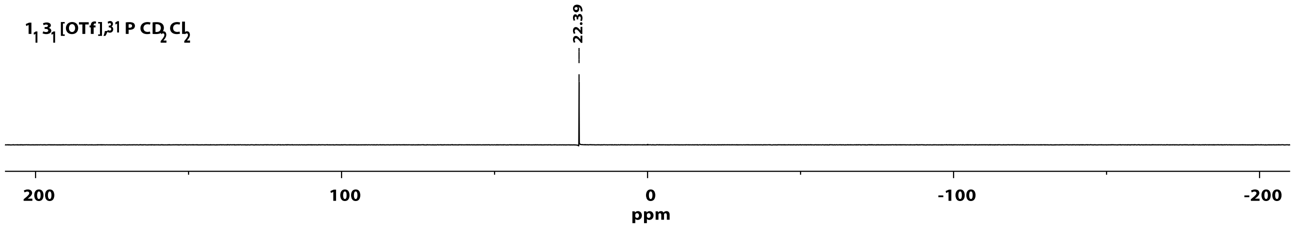
$1_1 3_1$ [OTf],31 P CD_2Cl_2
22.39
200
100
0
-100
-200
ppm

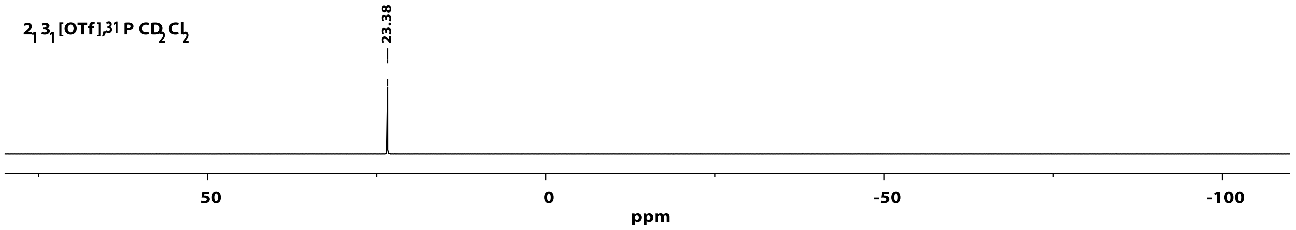
$2_1 3_1$ [OTf],31 P CD_2Cl_2
23.38
50
0
-50
-100
ppm

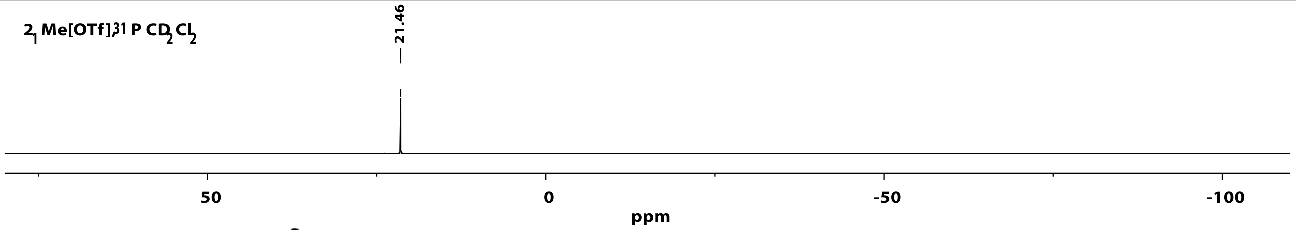
2$_1$ Me[OTf], 31 P CD_2Cl_2
21.46
50
0
-50
-100
ppm

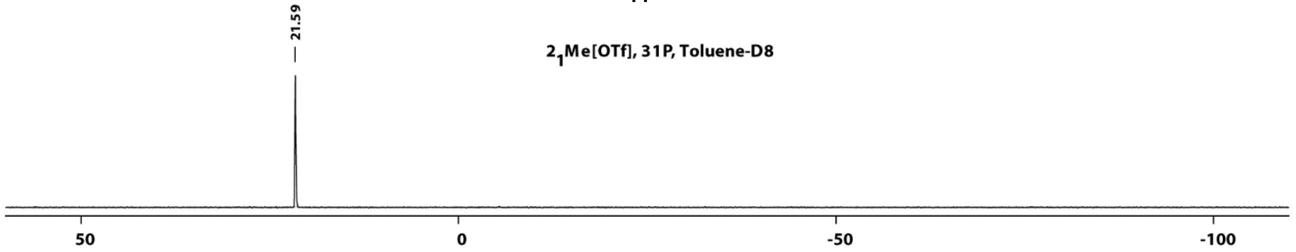
21.59
2$_1$Me[OTf], 31P, Toluene-D8
50
0
-50
-100

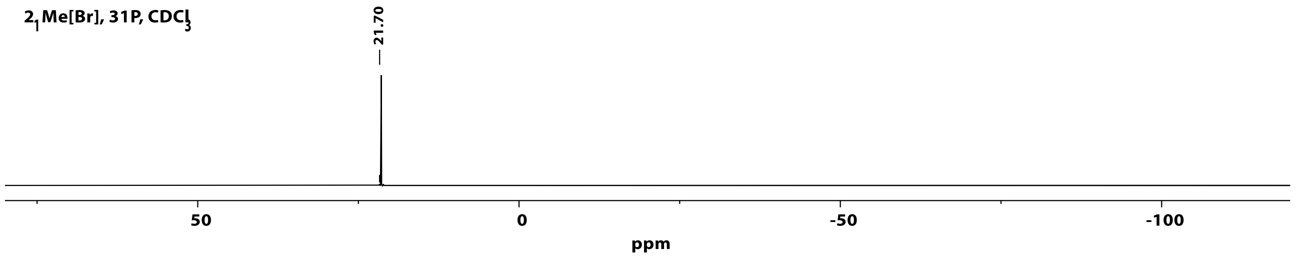
2$_1$ Me[Br], 31P, $CDCl_3$
21.70
50
0
-50
-100
ppm

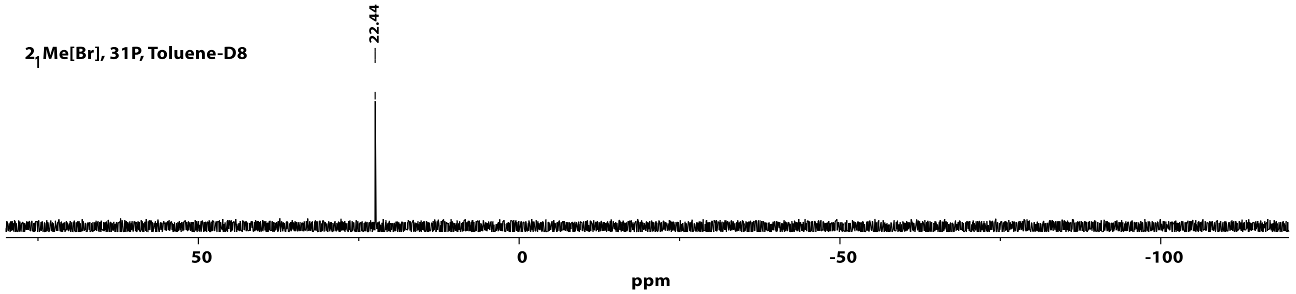
22.44
2$_1$ Me[Br], 31P, Toluene-D8
50
0
-50
-100
ppm

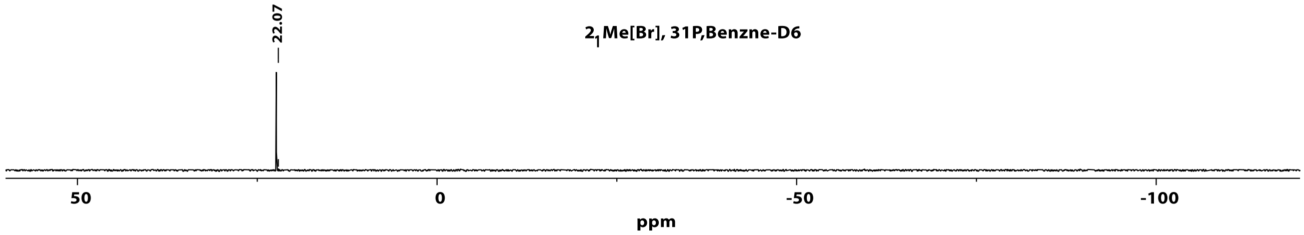
22.07
2$_1$ Me[Br], 31P,Benzne-D6
50
0
-50
-100
ppm

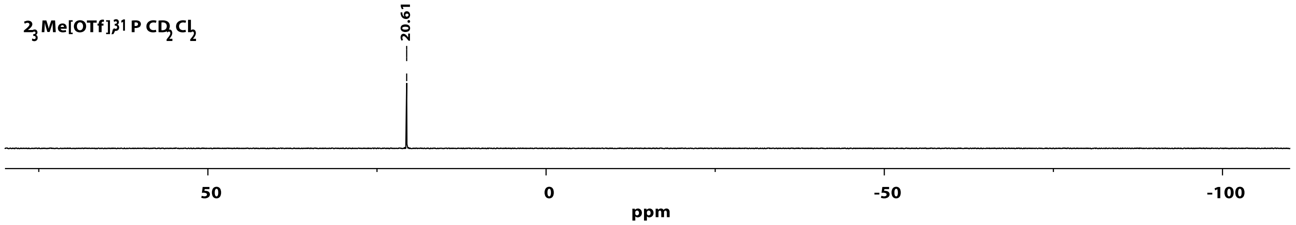
2$_3$ Me[OTf], 31 P CD_2Cl_2
20.61
50
0
-50
-100
ppm

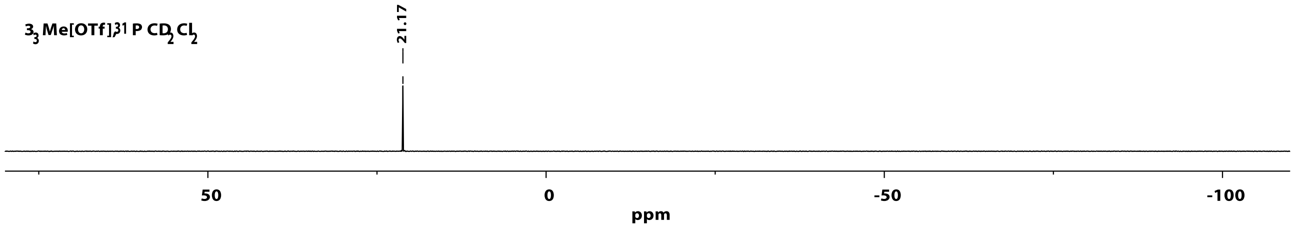
3$_3$ Me[OTf], 31 P CD_2Cl_2
21.17
50
0
-50
-100
ppm

References

[1] X. Yang, Y. Zhao, X. Zhang, R. Li, J. Dang, Y. Li, G. Zhou, Z. Wu, D. Ma, W.-Y. Wong, X. Zhao, A. Ren, L. Wang, X. Hou, *J. Mater. Chem.* **2012**, *22*, 7136-7148.
[2] Z. H. Li, M. S. Wong, Y. Tao, M. D'Iorio, *J. Org. Chem.* **2004**, *69*, 921–927.
[3] A. Belyaev, S. O. Slavova, I. V. Solovyev, V. Sizov, J. Jänis, E. V. Grachova, I. O. Koshevoy, *Inorg. Chem. Front.* **2020**, *7*, 140-149.
[4] I. Kondrasenko, Z.-H. Tsai, K.-y. Chung, Y.-T. Chen, Y. Y. Ershova, A. Doménech-Carbó, W.-Y. Hung, P.-T. Chou, A. J. Karttunen, I. O. Koshevoy, *ACS Appl. Mater. Interfaces* **2016**, *8*, 10968−10976.
[5] A. Belyaev, Y.-H. Cheng, Z.-Y. Liu, A. J. Karttunen, P.-T. Chou, I. O. Koshevoy, *Angew. Chem. Int. Ed.* **2019**, *58*, 13456–13465.
[6] a) J. P. Perdew, K. Burke, M. Ernzerhof, *Phys. Rev. Lett.* **1996**, *77*, 3865-3868; b) C. Adamo, V. Barone, *J. Chem. Phys.* **1999**, *110*, 6158-6170; c) M. A. Rohrdanz, K. M. Martins, J. M. Herbert, *J. Chem. Phys.* **2009**, *130*, 054112.
[7] F. Weigend, R. Ahlrichs, *Phys. Chem. Chem. Phys.* **2005**, *7*, 3297-3305.
[8] a) K. Eichkorn, O. Treutler, H. Öhm, M. Häser, R. Ahlrichs, *Chem. Phys. Lett.* **1995**, *240*, 283-290; b) M. Sierka, A. Hogekamp, R. Ahlrichs, *J. Chem. Phys.* **2003**, *118*, 9136-9148; c) F. Weigend, *Phys. Chem. Chem. Phys.* **2006**, *8*, 1057-1065.
[9] a) F. Furche, D. Rappoport, in *Computational Photochemistry* (Ed.: M. Olivucci), Elsevier, Amsterdam, **2005**, pp. 93-128; b) F. Furche, R. Ahlrichs, *J. Chem. Phys.* **2002**, *117*, 7433-7447.
[10] C. Holzer, *J. Chem. Phys.* **2020**, *153*, 184115.
[11] a) R. Ahlrichs, M. Bär, M. Häser, H. Horn, C. Kölmel, *Chem. Phys. Lett.* **1989**, *162*, 165-169; b) *TURBOMOLE* TURBOMOLE GmbH, **2020**.
